# Identification of genes involved in the ACC-mediated control of root cell elongation in *Arabidopsis thaliana*

**DOI:** 10.1186/1471-2229-12-208

**Published:** 2012-11-07

**Authors:** Marios Nektarios Markakis, Tinne De Cnodder, Michal Lewandowski, Damien Simon, Agnieszka Boron, Daria Balcerowicz, Thanaa Doubbo, Ludivine Taconnat, Jean-Pierre Renou, Herman Höfte, Jean-Pierre Verbelen, Kris Vissenberg

**Affiliations:** 1 Biology Dept., Plant Growth and Development, Univ. Antwerp, Groenenborgerlaan 171, Antwerpen, 2020, Belgium; 2 Unité Mixte de Recherche de Genomique Végétale, Institut National pour la Recherche Agronomique/Centre National pour la Recherche Scientifique, 2 rue Gaston Crémieux-CP 5708. F–91057, Evry Cedex, France; 3 Institut de Recherche en Horticulture et Semences UMR1345 (INRA/Agrocampus-ouest/Université d’Angers), Centre Angers-Nantes/INRA-IRHS batiment B, 42 rue Georges Morel – BP 60057 49071, Beaucouzé cedex, France; 4 Institut Jean-Pierre Bourgin, UMR1318 INRA-AgroParisTech, INRA Centre de Versailles-Grignon, Route de St-Cyr (RD10), F–78026, Versailles Cedex, France

**Keywords:** ACC, *Arabidopsis thaliana*, Development, Elongation control, Ethylene, Microarray analysis, Root growth

## Abstract

**Background:**

Along the root axis of *Arabidopsis thaliana*, cells pass through different developmental stages. In the apical meristem repeated cycles of division increase the numbers of cells. Upon leaving the meristem, these cells pass the transition zone where they are physiologically and mechanically prepared to undergo subsequent rapid elongation. During the process of elongation epidermal cells increase their length by 300% in a couple of hours. When elongation ceases, the cells acquire their final size, shape and functions (in the differentiation zone). Ethylene administered as its precursor 1-aminocyclopropane-1-carboxylic acid (ACC) is capable of inhibiting elongation in a concentration-dependent way. Using a microarray analysis, genes and/or processes involved in this elongation arrest are identified.

**Results:**

Using a CATMA-microarray analysis performed on control and 3h ACC-treated roots, 240 differentially expressed genes were identified. Quantitative Real-Time RT-PCR analysis of the 10 most up and down regulated genes combined with literature search confirmed the accurateness of the analysis. This revealed that inhibition of cell elongation is, at least partly, caused by restricting the events that under normal growth conditions initiate elongation and by increasing the processes that normally stop cellular elongation at the end of the elongation/onset of differentiation zone.

**Conclusions:**

ACC interferes with cell elongation in the *Arabidopsis thaliana* roots by inhibiting cells from entering the elongation process and by immediately stimulating the formation of cross-links in cell wall components, diminishing the remaining elongation capacity. From the analysis of the differentially expressed genes, it becomes clear that many genes identified in this response, are also involved in several other kind of stress responses. This suggests that many responses originate from individual elicitors, but that somewhere in the downstream signaling cascade, these are converged to a ’common pathway’. Furthermore, several potential keyplayers, such as transcription factors and auxin-responsive genes, were identified by the microarray analysis. They await further analysis to reveal their exact role in the control of cell elongation.

## Background

Plants are sessile organisms that are continuously impacted by changes in the environment. As a response a multitude of signal transduction cascades controls the development and metabolism of the plant such that it is continuously adapted to match the environmental challenges. In many instances it is the extent and direction of growth that is changed. Plant growth results from the formation of new cells during division and from the subsequent massive increase in volume during expansion of these newly formed cells. Ongoing research on both processes mainly exploits the model plant *Arabidopsis thaliana*, which represents one of the best experimental systems to study developmental processes in higher plants. Its whole genome is sequenced [1], the development occurs in a highly predictive and well-defined pattern [2] and cellular growth in the stem or the root can be easily monitored by means of microscopy [3]. Along the root axis, cells pass through different developmental stages. In the apical meristem, near the root tip, new cells are continuously formed by repeated cycles of division. Upon leaving the meristem, these cells pass the transition zone where they are physiologically and mechanically prepared to undergo rapid elongation. In the zone of rapid elongation these cells increase their length and volume by 300% in three hours. In the adjacent differentiation zone, the cells acquire their final shape and functions. At the root surface this can be seen as the emergence of root hairs on specific epidermal cells, the trichoblasts [4].

The massive increase in cell volume contributes substantially to the growth of plants. During the expansion, be it along several (e.g. leaf cells) or only one axis (e.g. root and hypocotyl cells), the cell wall is a centre of activity. Cellulose is the main constituent of the primary cell wall of vascular plants and forms the load-bearing network together with tethering xyloglucans (the main hemicellulose in plants like Arabidopsis). This network is laid down in a highly hydrophilic matrix, which contains pectins and structural proteins like arabinogalactan proteins (AGPs) and hydroxyproline-rich glycoproteins (HRGPs) [5]. Cellular growth results from the spatial separation of cellulose microfibrils, which requires modifications of the interconnecting xyloglucans and a force that pushes the microfibrils apart. The former is done by several classes of cell wall remodeling proteins, such as expansins [6] and xyloglucan endotransglucosylase/hydrolases (XTHs; [7,8]), both of which are proven to loosen walls, and by different enzyme activities as described in Frankova et al. [9]. The latter is provided by turgor pressure generated inside the cell. The process of cell expansion/elongation is highly complex and needs tight control as cell lysis by excessive turgor pressure or too loose walls needs to be prevented at all time. As mentioned, several players are known to contribute to this process, but the mechanism by which hormones and stressors exert control remains partly elusive.

In previous work it was shown that the gaseous plant hormone ethylene, administered as its precursor 1-aminocyclopropane-1-carboxylic acid (ACC), can reduce cell elongation in a concentration-dependent manner [3]. On the cellular level this inhibition is irreversible, root cells that ceased elongation cannot regain this. At the root level on the other hand, removal of ACC leads to normal elongation of these cells that are newly formed in the ACC-free condition. Some answers to the question how ethylene/ACC controls the maximal cell size in roots are found in the published literature and will be briefly discussed here. It is broadly accepted that for normal expansion to occur, expansins need a slightly acidic environment [10]. It is documented that ethylene/ACC exerts its effect on cell size by altering the auxin content in specific cells in the treated roots by modifying auxin transport and/or bio-synthesis [11]. As a result plasma membrane H^+^-ATPases are locked in their low-activity state, leading to an alkalinisation of cell walls instead of acidification and interfering with expansin-driven weakening of the walls [12]. At the same time peroxidase-mediated cross-linking activity in the cell wall further prevents cell expansion [13], resulting in the observed cell elongation phenotype. Since interference with the alkalinisation [12] or cross-linking activity [13] never restores growth to 100%, this clearly indicates that other yet to be discovered actors are at play. This study represents an attempt to reveal new actors in the control of cell elongation.

We will reveal differential gene expression levels between control and 3h ACC-treated Arabidopsis roots using CATMA microarray analysis, validate these by quantitative PCR analysis of some of the most altered genes and discuss the ethylene-mediated control of cell expansion. It is striking that genes coding for known cell wall loosening actors are down regulated, and genes coding for specific cell wall components together with their cross-linking enzymes are upregulated. The analysis of the 240 differentially expressed genes reveals that many genes identified in this ACC-evoked response, are also involved in other stress responses. This suggests that many responses may originate from individual elicitors, but that they may converge to a ’common pathway’ further downstream. Moreover, this microarray analysis identified several potential keyplayers, such as transcription factors and auxin-responsive genes, that await further analysis to reveal their exact role in the control of cell elongation.

## Results and discussions

### Changes in gene expression associated with the ACC-induced elongation arrest

We have previously shown that upon 5μM ACC addition the elongation of root epidermal cells is severely reduced in *Arabidopsis thaliana *[3]. To monitor changes in gene expression associated with the elongation-arrest, roots were harvested after 3h of ACC treatment. CATMA microarray analysis [14,15] was performed on three independent biological replicates. To determine differentially expressed genes between the two groups, a paired *t*-test was performed on the normalized log ratios of the fluorescence intensities. A total of 240 genes (0.98% of the total GSTs on the array) were significantly expressed (Bonferroni p-value cut-off 5%) between the two conditions. Out of the total number of genes, 166 were up regulated and 74 were down regulated by the ACC treatment. Additional file 1 represents all the differentially expressed genes. Quantitative PCR analysis was achieved for 19 of the 20 most affected genes (Table 1). The results of this analysis are depicted in Additional file 2 and they confirmed the outcome of the microarray; one, At5g25340, unfortunately failed to amplify.

**Table 1:** Top 10 of up (A) and down regulated (B) genes in 3h ACC-treated roots

**AGInr**	**Annotation**	**ratio**
AT3G59900	Auxin-Regulated Gene Involved in Organ Size (ARGOS)	3.61
AT5G19890	Peroxidase (PER59)	2.72
AT2G44080	ARGOS-LIKE	2.15
AT5G53980	Arabidopsis thaliana HomeoBox protein (ATHB52)	2.07
AT5G20820	SAUR-like auxin-responsive protein family (SAUR 75)	1.99
AT5G25340	Ubiquitin-like expressed protein	1.86
AT2G39980	HXXXD-type acyl-transferase family protein	1.83
AT4G28050	TET7 Member of TETRASPANIN family	1.75
AT1G49570	Peroxidase (PER10)	1.65
AT4G34110	Poly(A) Binding 2 (PAB2)	1.64
**AGInr**	**Annotation**	**ratio**
AT3G18000	Methyltransferase/ phosphoethanolamine N-methyltransferase XPL1 (XIPOTL 1)	−1.78
AT4G01630	Expansin (EXP17)	−1.76
AT4G35100	Plasma membrane Intrinsic Protein (PIP2;7/8)	−1.61
AT1G64390	Glycosyl hydrolase 9C2 (endo-1,4-beta-glucanase 6)	−1.60
AT4G25250	Pectin methylesterase inhibitor (PMEI)	−1.56
AT2G18800	Xyloglucan endotransglucosylase/hydrolase (XTH21)	−1.44
AT5G42590	Cytochrome P450 (CYP71A16)	−1.42
AT2G33790	Arabinogalactan protein (AGP30)	−1.38
AT3G25190	Nodulin-like 21	−1.37
AT4G28250	Beta-expansin (EXPB3)	−1.29

**Figure 1:**
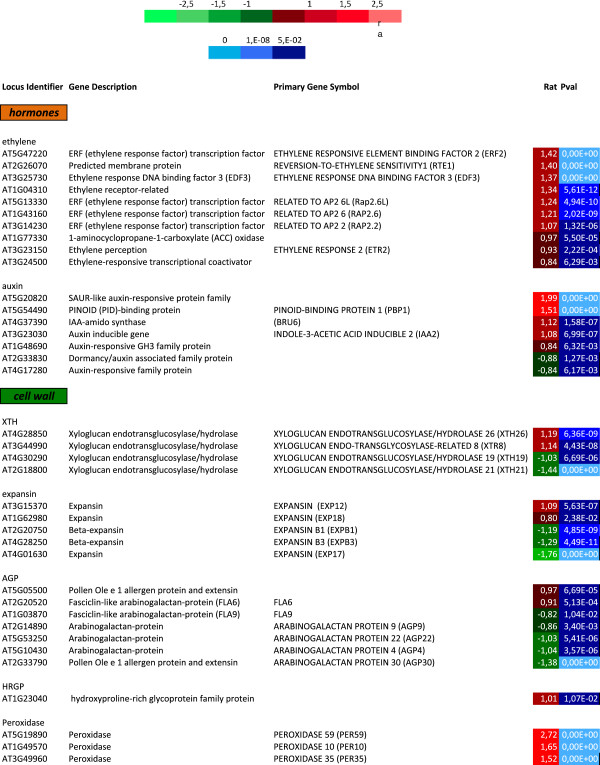
**Differentially expressed genes related to ethylene, auxin, XTH, expansin, AGP, HRGPs and peroxidases during ACC-induced inhibition of Arabidopsis root cell elongation.** The identified genes are presented with their locus identifier, the description of the gene, the primary gene symbol and with their expression ratio (color coded with bright green being the most down regulated and bright red the most up regulated) and Bonferroni P-values (color coded with bright blue representing a value of 0, dark blue a value between 1,E-08 and 5,E-02).

In theory, inhibition of cell elongation can be caused by restricting the events that under normal growth conditions initiate elongation, or by promoting the processes that normally stop cellular elongation at the end of the elongation/onset of differentiation zone. As explained before a reduction in cell wall loosening capacity or an increase in the cross-linking events can indeed partly account for the observed ACC-induced root phenotype. Therefore, the differentially expressed genes related to ethylene, auxin, XTH, expansin, AGP, HRGPs and peroxidases are presented in Figure 1 together with their expression ratio and p-values.

*A priori*, one would expect an ACC-induced upregulation of genes that under normal conditions display a higher expression at the end of the elongation zone, or at the onset of the differentiation zone, implying that they are somehow involved in the ending of the elongation process, or the onset of differentiation. Similarly, one would expect an ACC-induced down regulation of genes that are normally highly expressed at the start of or early during the elongation phase itself. To make the interpretation of the microarray results easier, the gene expression pattern was visualised using the Arabidopss eFP browser (http://bar.utoronto.ca/efp/cgi-bin/efpWeb.cgi; [16]) and presented in Additional file 3.

All the ethylene-related genes identified by the microarray are upregulated (Figure 1) and there does not seem to be a common expression pattern under normal conditions (Additional file 3A). Expression of specific genes was upregulated in a development-specific manner in trichoblasts, elongating cells and cells that have ceased to elongate.

Two out of 7 auxin-related genes that were identified by the microarray were down regulated. As auxin biosynthesis/transport is altered by ACC-addition to roots [11], it is not surprising that auxin-responsive genes become differentially regulated. For most genes, the change in expression level correlates well with their initial location of expression (Additional file 3B), being enriched at the onset or at the end of the elongation zone. This analysis identified several transcription factors and one small auxin upregulated (SAUR) gene. Mutant analysis will reveal the importance of changed expression patterns and the effect on their downstream targets.

For the cell-wall-loosening protein families expansin and XTH, the picture is rather less clear as some members are up regulated, whereas others are down regulated. The Additional file 1: Figure S1 in [12], however, shows the Arex database-extracted expression data of the expansin and XTH family and explains this seemingly contradictory behavior. For expansins it appears that the down regulated members (EXP17, EXPB1 and EXPB3) have an enriched expression in the expansion zone, whereas the expression of up regulated members (EXP12 and EXP18) normally starts at the end of the elongation zone and even preferentially in trichoblasts or root hair cells. As a secondary effect of ethylene addition is the ectopic formation of root hairs [17], it is plausible that we picked up those genes involved in the cell wall modifications necessary for this extra root hair formation (that are, however, not yet visible after 3 hours of ACC treatment). XTH26 is expressed in trichoblasts from the differentiation zone on, whereas XTH8 is enriched in the meristem and the elongation zone. XTH19 is ubiquitously expressed [18] and XTH21 highly expressed in the elongation zone. The down regulation of the last 2 XTHs can be linked to a potential reduction of the cell wall loosening capacity by ACC addition. In this respect, it is important to note that *xth21* mutants exhibit shorter roots [19]. The up regulated XTHs pose a problem in these terms, but it is clear that different members of the XTH family can have distinct characteristics making it difficult to generalize the function of isozymes [20,21].

As hypothesised in [13] and [12] ACC-induced cross-linking events could prevent cell elongation. Expression profiles of arabinogalactan proteins (AGPs), extensins and hydroxy-proline rich glycoproteins (HRGPs) indeed confirm that genes that are normally expressed from the end of the elongation zone on, are ACC-induced, whereas the ones expressed in actively expanding cells are down regulated by ACC (Additional file 3C).

As cross-linking of cell wall components can be altered in response to ACC, it seems that the peroxidases, mediating this reaction [22], are mimicking this behavior (Additional file 3D); two of them are normally enriched at the end of elongation, the third one is trichoblast-specific. According to Genevestigator (https://www.genevestigator.ethz.ch) their expression is significantly increased in response to stress, especially drought, UV-B light and wounding.

**Figure 2:**
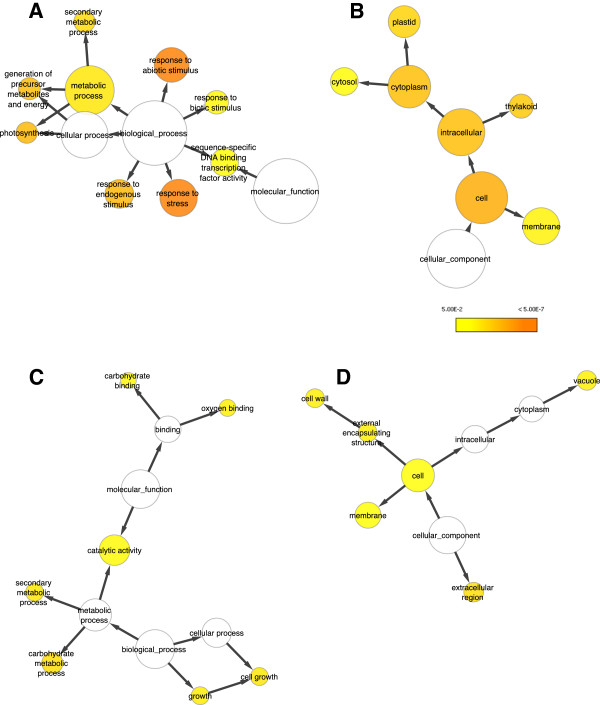
**Overrepresented functions and processes in 240 differentially expressed genes during ACC-induced inhibition of root cell elongation.** Gene Ontology (GO) categories that were statistically overrepresented (hypergeometric test, multiple testing correction - Benjamini & Hochberg False Discovery Rate (FDR) correction significance level 0.05) in the set of genes were represented as circles, their size relating to the number of associated genes, with p-values indicated by heat map colour. Linking arrows indicate GO subcategories within parent categories. Created in Cytoscape using the BiNGO plugin. (**A,B**) up regulated genes, (**C,D**) down regulated genes.

In the cluster of up regulated genes, enriched Gene Ontology (GO) terms (*p* ≤ 0.05) included response to abiotic stimuli (*P-*value 5.2807E-12), to stress (*P-*value 9.3374E-11) and to endogenous stimuli (*P-*value 3.8322E-6)(Figure 2A). Also enriched were response to biotic stimuli (*P-*value 4.6663E-3) and sequence-specific DNA binding transcription factor activity (*P-*value 7.4299E-3). In the down regulated cluster especially cell growth seemed overrepresented (*P-*value 3.8924E-4) (Figure 2C).

**Figure 3:**
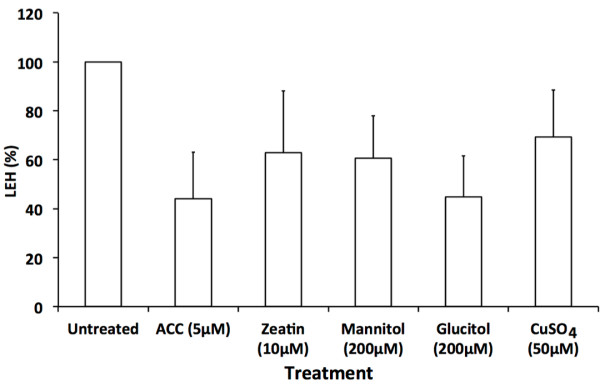
**Root growth in response to several stressors.** LEH of untreated roots and roots treated during 3 hours with 5μM ACC, 10μM zeatin, 200μM mannitol, 200μM glucitol or 50μM CuSO_4_ (means ± SD, in triplicates). Values after all treatments are statistically significant versus the untreated Col-0 (*t*-test, p<0.05, n>10).

Depending on the parameters used in the Cytoscape programme, a more complex network can be generated where overrepresented biological functions are more detailed (Additional file 4). From this it becomes clear that many up regulated genes are involved in all kinds of stresses, from water, osmotic, temperature and metal stress to responses towards auxin and abscisic acid (ABA). As similar genes seem to become differentially expressed in responses evoked by different elicitors, we investigated Arabidopsis root elongation under some of the aforementioned stress conditions (Figure 3). Elongation is evaluated by the length of the first epidermal cell with a visible root hair bulge (LEH), a marker for cell elongation in Arabidopsis roots [3]. From this figure it becomes clear that all kinds of stresses result in an inhibition of cell elongation, but all to a different extent. As a consequence, we can conclude that several stressors use pathways that converge to common genes to influence plant growth and behavior.

Besides focusing on ethylene, auxin and cell wall related genes, the 10 most up and down regulated genes in the microarray were identified together with their annotation and log_2_ ratio of expression between ACC treatment and control situation (Table 1).

Table 1 contains some genes that were not presented in Figure 1 and they will be discussed in the following paragraphs. Their expression patterns were extracted from the Arabidopsis eFP browser and are shown in Additional file 3E and Additional file 3F (for up and down regulated genes respectively).

### 10 most up regulated genes

The most up regulated gene identified by the microarray is the ARGOS gene (Auxin-Regulated Gene Involved in Organ Size), which was shown to control aerial organ size by influencing the number of cells, and not the size of cells. Further experiments suggested that this gene provides a link between hormone control and cell cycle activity through ANT and CYCD3;1 [23]. Besides expression in the aerial organs, ARGOS was also detected in the root tip and the pericycle, but no root phenotype was observed in plants with experimentally induced changes in expression levels [24]. As ACC/ethylene stimulate auxin biosynthesis and transport [11], it makes sense that upon addition, a highly auxin-responsive gene like ARGOS was detected in the microarray. A gene that is very closely related to ARGOS, the ARGOS-like (ARL) gene, appears third in our microarray list. From this gene it is known that over expression or reduced expression is positively correlated with cell size [25]. Amongst other genes it influences the expression of TCH4 [23], now known as xyloglucan endotransglucosylase/hydrolase (XTH) 22, which is expressed in roots (see suppl. data in [12]). The ARL gene is not only up regulated by ACC through auxin, but seems to be involved in brassinosteroid-dependent regulation of cell expansion [25]. In our detailed qPCR analysis it is the most upregulated in response to ACC, mainly due to its very low expression level in control conditions. So why is ARL up regulated to control inhibition of cell expansion? As ARL expression is correlated with expansion, we cannot rule out that in the ACC response its positive effect is overruled by other processes that reduce cell expansion.

The ACC-induced Arabidopsis Thaliana HomeoBox protein 52 (ATHB52) is a plant-specific homeodomain leucine zipper Class I transcription factor family member. This family has been reported to play an important role in photomorphogenesis and de-etiolation, and to be affected by light conditions [26] and auxin (Genevestigator, [27]). These transcription factors typically need to dimerize before exerting their function on DNA. Functional characterization of this gene/protein is missing at the moment, but transgenic plants expressing other family members, *ATHB1*, -*3*, -*6*, -*7*, -*12*, -*13*, -*16*, -*20*, or −*23* at increased levels all show a reduction in cell expansion in different organs and with somewhat different consequences for organ development [26]. Furthermore, the expression pattern in the root, as extracted from the Arex database [16], is highest at the boarder between the elongation and the differentiation zone. From these data it can be postulated that this gene fulfils a crucial role in the control of cellular elongation, making it interesting to see which downstream genes are influenced by ATHB52.

There is not much information on AT5G25340, which encodes a Ubiquitin-like expressed protein. Vergnolle et al. [28] reported it to be up regulated by cold-treatment, downstream of Phospholipase C and D activity in Arabidopsis cell suspensions, and according to Genevestigator [27] it is up regulated by Methyl-Jasmonate. Effects of synthetic jasmonates include inhibition of stem and root growth [29], moreover MeJa is associated with stress [30] making it possible that this gene is involved in the cross-talk of Meja and ethylene. Ubiquitin and small ubiquitin-like modifiers (UBLs) are generally small proteins (SUMO; AT5G25340 consists of 208 amino acids) that covalently modify other proteins and thereby alter the activity of many substrate proteins [31,32].

Few data are available on HXXXD-type acyl-transferase family protein (AT2G39980). It has been reported to be up regulated in microarray analyses of cross-talk between jasmonic acid and ethylene signaling in Arabidopsis seedlings, of heat shock treatment, and of early post germination embryos treated with paclobutrazol and ABA (Genevestigator, [27]).

The mRNA level of a senescence-associated member of the TETRASPANIN family was differential between control and ACC-treated roots. Previous reports identified a tetraspanin-related signalling pathway that interacts with auxin-related processes, based on mutants with patterning defects in leaves and in the root epidermis [33,34]. Tetraspanins are only present in multicellular organisms and they interact with one another and with other transmembrane proteins to facilitate ligand binding, signalling downstream of associated proteins, cell-to-cell adhesion or fusion and proteolysis. As ethylene triggers a signal cascade, the up regulation of this gene could increase signalling events.

The 10th most upregulated gene in response to ACC treatment is the Poly(A) binding protein 2, an important translation initiator factor which has been shown to interact with the RNA-dependent RNA polymerase (RdRp) and the viral genome-linked protein (VPg-Pro) of turnip mosaic virus [35,36] and references herein. As ethylene is associated with pathogen infection [37], one of the outcomes of the pathway is the increase in this gene. The role it plays in the inhibition of cell elongation is not clear.

### 10 most down regulated genes

Our data identifies XPL1, coding for a methyltransferase with a key role in the biosynthesis of phosphatidylcholine, the major lipid component in plant cell membranes, as the highest down regulated gene by ACC. Mutants in XPL1 show significantly shorter primary roots, more lateral roots, drastically fewer root hairs and short epidermal cells with aberrant morphology and increased cell death [38]. These phenotypes were probably not only due to the lack of the cell membrane component itself, but also to the subsequent lack of PA (phosphatidic acid), which has an important role in signalling pathways. The mutant short root phenotype matches with the ACC-induced root response and identifies lipid metabolism as an important regulatory mechanism for cell elongation.

Next in the table is PIP2;7/8, which is a plasma membrane intrinsic protein functioning as an aquaporin [39,40]. Down regulation of this gene can have important consequences for cellular growth, since this is governed by vacuole-driven uptake of water, and especially on responses to different stresses [41]. As ACC addition can be regarded as a signal for stress [42] a down regulation of this gene could change the potential of the cell to provide a pushing force to the cell wall, needed for cell expansion to occur. Its high expression in expanding cells strengthens this hypothesis (Additional file 3F).

A glycosyl hydrolase 9C2, known as endo-1,4-beta-glucanase 6 [43], is down regulated by ACC. No direct biological function is proven for this particular gene, but other members of this 25-gene family are involved in cellulose biosynthesis or wall deposition and integration (KOR1 [44], KOR2 and KOR3 [45], AtCEL1 [46], AtCEL5 [47]). This gene contains an N-terminal signal peptide targetting it to the wall, a glycosyl hydrolase family 9-specific catalytic domain and a family 49 carbohydrate binding module [43]. If the gene is indeed involved in cell wall construction, it is plausible that inhibition of cell elongation also means that no extra (cellulosic) cell wall components need to be synthesized, hence resulting in the down regulation of this gene. Whether the down regulation is the consequence or cause of the failure to elongate remains to be uncovered. It is known that inhibition of cellulose synthesis, in mutants or by chemical interference, negatively regulates normal cell elongation.

Another cell wall-related gene is AT4G25250, which codes for a pectin methylesterase inhibitor (PMEI), which is highly expressed only in the root elongation zone (Additional file 3F). This class of inhibitors regulates the activity of pectin methylesterases (PME) that modify the level of methylesterification of pectins. Normally pectins are synthesized in a highly methyl- and acetylesterified form. During cellular development, PMEs cleave the methylesters to reveal carboxylic groups [48], which are prone to interact with divalent cations like Ca^2+^, glueing together two adjacent pectin-chains in so-called egg-box formation. This ’cross-linking’ of pectins can reduce pore size in the cell wall, preventing cell wall modifying enzymes from freely moving around, and can therefore also interfere with cell expansion [49,50]. Besides creating egg-box domains, the carboxylic groups are acidic, changing the environmental pH for enzymes acting on cell wall components [51]. In combination with the expression data, the enzymatic activity and its consequences point to a pivotal role in the control of cell elongation.

Not much is known on the function of At5g42590, a putative cytochrome P450 with an enriched expression at the onset of the elongation and differentiation zone. P450s are encoded by a highly divergent gene superfamily containing 256 members, which carry out a wide diversity of reactions [52], making it difficult to suggest a specific function for this gene.

The nodulin-like family encodes presumed membrane proteins with five calculated transmembrane domains and significant protein sequence homology to the vacuolar iron transporters. Additional to our data it was shown that *nodulin-like21* mutants had significantly decreased Fe in roots, but no apparent root phenotype, and that the gene was down regulated in response to Fe deficiency [53].

## Conclusions

ACC interferes with cell elongation in *Arabidopsis thaliana* roots by inhibiting cells from initiating the elongation process. This is seen in the down regulation of cell wall loosening proteins, plasma membrane aquaporins that transport water across the plasma membrane and aid in the generation of turgor pressure, pectin modifying enzymes and lipids involved in signalling events. On top of the repression of the elongation start, the inhibition of elongation is stimulated by increasing the formation of specific cell wall components and their cross-linking enzymes. Many genes identified in this elongation arrest response, are also involved in several other stress responses, suggesting that somewhere in the downstream signaling cascade, these responses converge to a ’common pathway’. Moreover, several potential keyplayers, such as transcription factors and auxin-responsive genes, were identified by the microarray analysis. Mutant analysis will reveal their exact role in the control of cell elongation.

## Methods

### Plant material and growth conditions

*Arabidopsis thaliana* Col-0 wild type seeds were obtained from the Nottingham Stock centre. They were surface-sterilized in 6% (v/v) commercial bleach for 15 min and rinsed 5 times with distilled water. All seeds were placed on a Murashige and Skoog (1/2 MS) medium including vitamins (Duchefa, The Netherlands), supplemented with 10g/L sucrose and solidified with 8g/L Gelrite (Duchefa, The Netherlands) at pH 5.7. After overnight incubation at 4°C, the dishes were placed vertically in a growth chamber at 22°C in a 16 h light / 8 h dark photoperiod at a light intensity of 24 *μ*mol/m^2^/s (PAR, Philips tlm 65W/33). Five-day-old seedlings were transferred to normal MS media (as a control for the transfer effects) or to media supplemented with 5μM 1-aminocyclopropane-1-carboxylic acid (ACC, Acros Organics). After 3h of growth, roots were harvested and the RNA was isolated.

For stress-related LEH measurements, 5-day-old seedlings were transfered to ½MS plates including 5μM ACC, 10μM zeatin, 200μM mannitol, 200μM glucitol or 50μM CuSO_4_. The LEH was measured with the freely available tool ImageJ (http://rsbweb.nih.gov/ij/) on digital images of the root apices made using a Nikon DXM1200 digital camera mounted on a Zeiss Axioskope.

### RNA isolation and microarray analysis

The roots were cut from the seedlings and immediately frozen in liquid nitrogen prior to grinding with a mortar and a pestle. The total RNA of at least 500 pooled roots was isolated using the Rneasy Plant Mini kit (Qiagen) following the supplied manual. A minimum quantity of 4 μg total RNA is required for further analysis. Microarray hybridizations for 3 independent biological repetitions (i.e. 500 roots each) were carried out with the CATMA array [14,15], which contains 24 576 gene-specific tags [54], corresponding to 22 089 genes as described in [55] and which is used in numerous other studies (e.g. [50]).

The raw data are the log of the median feature pixel intensity at wavelength 635 nm and 532 nm. The statistical analysis was mainly as described by [56] and was based on three dye swaps. The methods are available in the R package Anapuce (http://cran.r-project.org/web/packages/anapuce/index.html). The normalization and statistical analyses were based on three dye swaps (i.e. six arrays) per comparison. First, one normalization without background substraction is performed to remove systematic biases. Then, a global intensity dependent normalization is performed using the lowess procedures to correct the dye bias. Finally, for each block, the log-ratio median calculated over the values for the entire block is subtracted from each individual log ratio value to correct effects on each block, as well as print-tip, washing and/or drying effects. To determine differentially expressed genes from a dye-swap, a paired t-test is performed on the log2 ratios, with a common variance for all the genes (H homoscedasticity), leading to a robust estimation of the variance and a high power of the test. Spots with an extreme variance or genes or which only one observation is available are excluded. Then, the raw P-values are adjusted by the Bonferroni method, which controls the Family Wise Error Rate, genes with a Bonferroni P-value < 0.05 were considered differentially expressed, as described in [56]. In Figure 1 and Table 1 the log ratio (rat) refers to the differential expression level between control roots and roots treated with ACC, based on a paired *t*-test. A positive value of the ratio corresponds to an up regulation by ACC, whereas a negative value points to a down regulation by ACC.

Sequence data from this article were deposited in ArrayExpress (http:// http://www.ebi.ac.uk/arrayexpress/) according to the MIAME standards (accession no. E-MEXP-362) and in CATdb (http://urgv.evry.inra. fr/CATdb/; Project nr: CATMA-INRA05-01).

### RNA extraction and quantitative RT-PCR

RNA was extracted from approximately 20 root elongation zones with a length of 500μm, measured from the root tip, using the RNAqueous® kit (Life Technologies) and following the manufacturers protocol. The quantity of RNA was measured with a nanodrop ND 1000 (Thermo Scientific) and cDNA synthesis was performed with SuperScript TM II Reverse Transcriptase according to the provided protocol. Real time PCR was performed using TaqMan® Universal Master Mix II with UNG (Life Technologies). Multiplex PCR was performed with act 8 (Probe id At02270958_gH) as endogenous control and TaqMan® probes for each gene as specified in Table 2. Three technical and 4 to 5 biological repeats were performed. The results were analysed with the StepOnePlus™ Real-Time Software.

**Table 2:** Probes used to perform qPCR of the 10 most up and down regulated genes

Up regulated genes	Probe ID	Down regulated genes	Probe ID
AT3G59900	At02197257_s1	AT3G18000	At02253838_g1
AT5G19890	At02210962_g1	AT4G01630	At02207775_g1
AT2G44080	At02358564_s1	AT4G35100	At02255535_gH
AT5G53980	At02321432_s1	AT1G64390	At02218323_m1
AT5G20820	At02302302_s1	AT4G25250	At02238255_s1
AT5G25340	At02304437_g1	AT2G18800	At02177336_gH
AT2G39980	At02324966_s1	AT5G42590	At02314387_g1
AT4G28050	At02300938_g1	AT2G33790	At02204410_m1
AT1G49570	At02271157_g1	AT3G25190	At02280027_g1
AT4G34110	At02249725_g1	AT4G28250	At02301064_gH

## Abbreviations

ACC: Aminocyclopropane-1-carboxylic acid; AGP: Arabinogalactan protein; ARGOS: Auxin-regulated gene involved in organ size; ARL: Argos-like; ATHB: Arabidopsis thaliana homeobox protein; HRGP: Hydroxyproline-rich glycoprotein; LEH: Length of the first epidermal cell with a visible root hair bulge; MeJa: Methyl jasmonate; PME(I): Pectin methylesterase (inhibitor); XTH: Xyloglucan endotransglucosylase/hydrolase.

## Competing interests

The authors declare that they have no competing interests.

## Authors’ contributions

MNM, TDC, ML, DS, AB, DB, TD carried out the plant manipulations, RNA extraction, qPCR analysis, micro-array queries and bio-informatic analysis. LT, J-PR and HH carried out the micro-array analysis. J-PR, HH, J-PV and KV participated in the design of the study. MNM, TDC and KV drafted the manuscript. All authors read and approved the final manuscript.

## Supplementary Material

Additional file 1 **240 differentially expressed genes upon 3hr 5μM ACC treatment.** The genes (locus identifiers) are presented together with their expression ratio and Bonferroni P-values. Click here for file

Additional file 2 **qPCR analysis of the 10 most up and down regulated genes upon 3 hr 5μM ACC addition to Arabidopsis roots.** Expression is presented as relative % towards the gene’s expression under control conditions. Click here for file

Additional file 3 **Expression profiles in the root of the genes presented in Table **2**.** Data were extracted from the Arabidopsis eFP browser. A) ethylene-related genes, B) auxin-related genes, C) AGPs and HRGPs, D) peroxidases, E) 10 most up regulated genes, F) 10 most down regulated genes. Click here for file

Additional file 4 **Enriched Gene Ontology (GO) terms in the differentially expressed genes.** A) cluster of up regulated genes, B) cluster of down regulated genes, with the legend linking the numbers to the GI terms. Click here for file
