# Supplementary material for: Identification of genes involved in the ACC-mediated control of root cell elongation in Arabidopsis thaliana
Source: BMC Plant Biol. 2012 Nov 7;12:208. doi: 10.1186/1471-2229-12-208 (PMC3502322; doi:10.1186/1471-2229-12-208)
Supplement: Additional file 1 — 240 differentially expressed genes upon 3hr 5μM ACC treatment. The genes (locus identifiers) are presented together with their expression ratio and Bonferroni P-values. [file 1471-2229-12-208-S1.pdf]

Gene identifier	Function	Rat	Pval
AT3G59900	ARGOS	3,61	0,00E+0
AT5G19890	peroxidase, putative	2,72	0,00E+0
AT2G44080	ARGOS-like	2,15	0,00E+0
AT5G53980	homeobox-leucine zipper family protein	2,07	0,00E+0
AT5G20820	auxin-responsive protein-related	1,99	0,00E+0
AT5G25340	expressed protein	1,86	0,00E+0
AT2G39980	transferase family protein	1,83	0,00E+0
AT4G28050	senescence-associated protein, putative	1,75	0,00E+0
AT1G49570	peroxidase, putative	1,65	0,00E+0
AT4G34110	polyadenylate-binding protein 2 (PABP2)	1,64	0,00E+0
AT4G35160	O-methyltransferase family 2 protein	1,60	0,00E+0
AT3G49960	peroxidase, putative	1,52	0,00E+0
AT5G54490	calcium-binding EF-hand protein, putative	1,51	0,00E+0
AT2G41090	calmodulin-like calcium-binding protein, 22 kDa (CaBP-22)	1,50	0,00E+0
AT5G52640	heat shock protein 81-1 (HSP81-1) / heat shock protein 83 (HSP83)	1,49	0,00E+0
AT4G26220	caffeoyl-CoA 3-O-methyltransferase, putative	1,47	0,00E+0
AT5G14780	formate dehydrogenase (FDH)	1,47	0,00E+0
AT5G01210	transferase family protein	1,46	0,00E+0
AT2G43590	chitinase, putative	1,45	0,00E+0
AT2G42850	cytochrome P450 family protein	1,42	0,00E+0
AT5G47220	ethylene-responsive element-binding factor 2 (ERF2)	1,42	0,00E+0
AT5G59540	oxidoreductase, 2OG-Fe(II) oxygenase family protein	1,41	0,00E+0
AT2G22880	VQ motif-containing protein	1,41	0,00E+0
AT2G26070	RTE1 (REVERSION-TO-ETHYLENE SENSITIVITY1)	1,40	0,00E+0
AT3G33002	pseudogene, ribosomal protein S2p family	1,39	0,00E+0
AT5G40590	DC1 domain-containing protein	1,38	0,00E+0
AT3G25730	AP2 domain-containing transcription factor, putative	1,37	0,00E+0
AT1G18100	mother of FT and TF1 protein (MFT)	1,35	0,00E+0
AT4G01870	tolB protein-related	1,34	5,61E-12
AT1G04310	ethylene receptor-related	1,34	5,61E-12
AT2G38240	oxidoreductase, 2OG-Fe(II) oxygenase family protein	1,31	1,68E-11
AT4G34540	isoflavone reductase family protein	1,29	3,93E-11
AT5G51440	23.5 kDa mitochondrial small heat shock protein (HSP23.5-M)	1,26	2,30E-10
AT5G25450	ubiquinol-cytochrome C reductase complex 14 kDa protein, putative	1,25	3,98E-10
AT4G38410	dehydrin, putative	1,24	4,49E-10
AT4G02520	glutathione S-transferase, putative	1,24	4,60E-10
AT5G13330	AP2 domain-containing transcription factor family protein	1,24	4,94E-10
AT5G10770	chloroplast nucleoid DNA-binding protein, putative	1,23	7,58E-10
AT1G55920	serine O-acetyltransferase, putative	1,23	9,54E-10
AT5G06860	polygalacturonase inhibiting protein 1 (PGIP1)	1,22	1,25E-9
AT1G43160	AP2 domain-containing protein RAP2.6 (RAP2.6)	1,21	2,02E-9
AT4G24960	ABA-responsive protein (HVA22d)	1,21	2,12E-9
AT5G39050	transferase family protein	1,21	2,61E-9
AT1G79160	expressed protein	1,20	3,66E-9
AT4G28850	xyloglucan:xyloglucosyl transferase, putative / xyloglucan endotransglycosylase, putative / endo-xyloglucan transferase, putative	1,19	6,36E-9
AT4G36990	heat shock factor protein 4 (HSF4) / heat shock transcription factor 4 (HSTF4)	1,18	1,10E-8
AT1G61890	MATE efflux family protein	1,17	1,33E-8
AT1G07400	17.8 kDa class I heat shock protein (HSP17.8-CI)	1,15	3,94E-8
AT3G44990	xyloglucan:xyloglucosyl transferase, putative / xyloglucan endotransglycosylase, putative / endo-xyloglucan transferase, putative	1,14	4,43E-8
AT3G46230	17.4 kDa class I heat shock protein (HSP17.4-CI)	1,14	5,69E-8
AT5G02760	protein phosphatase 2C family protein / PP2C family protein	1,13	8,53E-8
AT2G41410	calmodulin, putative	1,13	9,31E-8
AT2G07671	H+-transporting two-sector ATPase, C subunit family protein	1,13	9,71E-8
AT1G75390	bZIP transcription factor family protein	1,12	1,15E-7
AT4G37390	auxin-responsive GH3 family protein	1,12	1,58E-7
AT1G78000	sulfate transporter (Sultr1;2)	1,12	1,60E-7
AT1G19900	glyoxal oxidase-related	1,11	1,62E-7
AT4G30430	senescence-associated family protein	1,11	1,87E-7
AT1G02850	glycosyl hydrolase family 1 protein	1,10	2,50E-7
AT1G77120	alcohol dehydrogenase (ADH)	1,10	2,55E-7
AT1G60010	expressed protein	1,10	2,89E-7
AT5G54160	quercetin 3-O-methyltransferase 1 / flavonol 3-O-methyltransferase 1 / caffeic acid/5-hydroxyferulic acid O-methyltransferase (OMT1)	1,10	3,40E-7
AT3G15370	expansin, putative (EXP12)	1,09	5,63E-7
AT4G02380	late embryogenesis abundant 3 family protein / LEA3 family protein	1,09	5,63E-7
AT2G36090	F-box family protein	1,09	5,67E-7
AT3G23030	auxin-responsive protein / indoleacetic acid-induced protein 2 (IAA2)	1,08	6,99E-7
AT1G67340	zinc finger (MYND type) family protein / F-box family protein	1,08	7,88E-7
AT2G47550	pectinesterase family protein	1,07	9,83E-7
AT3G14230	AP2 domain-containing protein RAP2.2 (RAP2.2)	1,07	1,32E-6
AT1G74100	sulfotransferase family protein	1,06	1,83E-6
AT5G52310	low-temperature-responsive protein 78 (LTI78) / desiccation-responsive protein 29A (RD29A)	1,05	2,31E-6
AT2G27580	zinc finger (AN1-like) family protein	1,05	2,78E-6
AT3G51410	expressed protein	1,04	3,93E-6
AT5G49480	sodium-inducible calcium-binding protein (ACP1) / sodium-responsive calcium-binding protein (ACP1)	1,03	6,09E-6
AT1G79470	inosine-5prim-monophosphate dehydrogenase	1,02	7,86E-6
AT5G63840	alpha-glucosidase, putative	1,02	8,01E-6
AT5G63790	no apical meristem (NAM) family protein	1,02	8,22E-6
AT3G48990	AMP-dependent synthetase and ligase family protein	1,02	8,74E-6
AT3G54040	photoassimilate-responsive protein-related	1,01	1,10E-5
AT3G03990	esterase/lipase/thioesterase family protein	1,01	1,21E-5

AT2G32270	zinc transporter (ZIP3)	1,01	1,36E-5
AT1G23040	hydroxyproline-rich glycoprotein family protein	1,01	1,07E-2
AT4G02450	glycine-rich protein	1,00	1,90E-5
AT3G07810	heterogeneous nuclear ribonucleoprotein, putative / hnRNP, putative	1,00	2,00E-5
AT3G47510	expressed protein	1,00	2,05E-5
AT3G24420	hydrolase, alpha/beta fold family protein	0,99	2,22E-5
AT3G55790	expressed protein	0,99	1,45E-2
AT4G34710	arginine decarboxylase 2 (SPE2)	0,99	2,50E-5
AT3G09350	armadillo/beta-catenin repeat family protein	0,99	2,50E-5
AT3G52340	sucrose-phosphatase 2 (SPP2)	0,99	2,53E-5
AT2G20560	DNAJ heat shock family protein	0,99	2,76E-5
AT5G64090	expressed protein	0,98	3,33E-5
AT1G77330	1-aminocyclopropane-1-carboxylate oxidase, putative / ACC oxidase, putative	0,97	5,50E-5
AT2G29440	glutathione S-transferase, putative	0,97	6,19E-5
AT5G05500	pollen Ole e 1 allergen and extensin family protein	0,97	6,69E-5
AT1G76470	cinnamoyl-CoA reductase family	0,96	7,17E-5
AT5G57660	zinc finger (B-box type) family protein	0,95	1,13E-4
AT4G14030	selenium-binding protein, putative	0,95	1,32E-4
AT3G01970	WRKY family transcription factor	0,94	1,87E-4
ATMG00990	NADH dehydrogenase subunit 3	0,94	1,89E-4
AT3G23150	ethylene receptor, putative (ETR2)	0,93	2,22E-4
AT5G17760	AAA-type ATPase family protein	0,93	2,41E-4
AT3G11690	expressed protein	0,93	2,84E-4
AT3G18200	nodulin MtN21 family protein	0,93	2,89E-4
AT2G18680	expressed protein	0,93	3,00E-4
AT3G28210	zinc finger (AN1-like) family protein	0,92	3,45E-4
AT3G14680	cytochrome P450, putative	0,92	3,61E-4
AT2G20520	fasciclin-like arabinogalactan-protein (FLA6)	0,91	5,13E-4
AT5G09440	phosphate-responsive protein, putative	0,91	5,15E-4
AT2G47270	expressed protein	0,91	5,39E-4
AT1G59740	proton-dependent oligopeptide transport (POT) family protein	0,90	6,97E-4
AT2G07675	ribosomal protein S12 mitochondrial family protein	0,90	7,37E-4
AT3G10070	transcription initiation factor IID (TFIID) subunit A family protein	0,90	8,45E-4
AT5G02550	expressed protein	0,89	9,19E-4
AT1G19530	expressed protein	0,89	9,71E-4
AT5G10625	expressed protein	0,89	1,07E-3
AT2G37970	SOUL heme-binding family protein	0,88	1,36E-3
AT5G03670	expressed protein	0,88	1,43E-3
AT2G47950	expressed protein	0,88	1,45E-3
AT2G37750	expressed protein	0,88	1,66E-3
AT4G24480	serine/threonine protein kinase, putative	0,87	2,03E-3
AT3G49940	LOB domain protein 38 / lateral organ boundaries domain protein 38 (LBD38)	0,87	2,03E-3
AT1G58170	disease resistance-responsive protein-related / dirigent protein-related	0,87	2,33E-3
AT5G62280	expressed protein	0,87	2,39E-3
AT2G36080	DNA-binding protein, putative	0,86	2,47E-3
AT5G25110	CBL-interacting protein kinase 25 (CIPK25)	0,86	2,66E-3
AT2G24260	basic helix-loop-helix (bHLH) family protein	0,86	2,70E-3
AT5G51830	pfkB-type carbohydrate kinase family protein	0,86	2,83E-3
AT2G47730	glutathione S-transferase 6 (GST6)	0,86	2,96E-3
AT1G10140	expressed protein	0,86	2,99E-3
AT1G76180	dehydrin (ERD14)	0,86	3,29E-3
AT5G13180	no apical meristem (NAM) family protein	0,85	3,65E-3
AT1G73480	hydrolase, alpha/beta fold family protein	0,85	4,03E-3
AT2G19310	expressed protein	0,85	4,05E-3
AT1G02900	rapid alkalinization factor (RALF) family protein	0,85	4,64E-3
AT1G78820	curculin-like (mannose-binding) lectin family protein / PAN domain-containing protein	0,85	4,79E-3
AT2G47170	ADP-ribosylation factor 1 (ARF1)	0,84	5,20E-3
AT3G02470	adenosylmethionine decarboxylase family protein	0,84	5,33E-3
AT5G10960	CCR4-NOT transcription complex protein, putative	0,84	6,04E-3
AT3G24500	ethylene-responsive transcriptional coactivator, putative	0,84	6,29E-3
AT1G48690	auxin-responsive GH3 family protein	0,84	6,32E-3
AT2G16480	SWIB complex BAF60b domain-containing protein / plus-3 domain-containing protein	0,84	6,42E-3
AT1G07350	transformer serine/arginine-rich ribonucleoprotein, putative	0,83	6,81E-3
AT2G30140	UDP-glucoronosyl/UDP-glucosyl transferase family protein	0,83	6,92E-3
AT1G51680	4-coumarate--CoA ligase 1 / 4-coumaroyl-CoA synthase 1 (4CL1)	0,82	9,80E-3
AT1G22280	protein phosphatase 2C, putative / PP2C, putative	0,82	1,08E-2
AT1G53560	expressed protein	0,81	1,29E-2
AT3G52870	calmodulin-binding family protein	0,81	1,35E-2
AT5G48150	phytochrome A signal transduction 1 (PAT1)	0,81	1,40E-2
ATMG01275	NADH dehydrogenase subunit 1	0,81	1,64E-2
AT4G13040	AP2 domain-containing transcription factor family protein	0,81	1,70E-2
AT5G54280	myosin heavy chain, putative	0,80	1,99E-2
AT5G06280	expressed protein	0,80	2,05E-2
AT1G06570	4-hydroxyphenylpyruvate dioxygenase (HPD)	0,80	2,27E-2
AT2G37220	29 kDa ribonucleoprotein, chloroplast, putative / RNA-binding protein cp29, putative	0,80	2,35E-2
AT1G62980	expansin, putative (EXP18)	0,80	2,38E-2
AT4G39780	AP2 domain-containing transcription factor, putative	0,80	2,39E-2
AT1G30760	FAD-binding domain-containing protein	0,79	2,52E-2
AT1G64720	expressed protein	0,79	2,58E-2
AT5G38200	expressed protein	0,79	2,87E-2
AT5G03380	heavy-metal-associated domain-containing protein	0,79	2,95E-2

AT3G45300	isovaleryl-CoA-dehydrogenase (IVD)	0,78	3,36E-2
AT3G01290	band 7 family protein	0,78	3,48E-2
AT5G14040	mitochondrial phosphate transporter	0,78	4,04E-2
AT4G12720	MutT/nudix family protein	0,78	4,19E-2
AT3G47540	chitinase, putative	0,77	4,71E-2
AT3G20810	transcription factor jumonji (jmjC) domain-containing protein	-0,77	4,89E-2
AT1G43580	expressed protein	-0,77	4,52E-2
AT5G42630	myb family transcription factor (KAN4)	-0,78	4,23E-2
AT2G29550	tubulin beta-7 chain (TUB7)	-0,79	2,85E-2
AT1G22880	glycosyl hydrolase family 9 protein	-0,79	2,66E-2
AT5G60520	late embryogenesis abundant protein-related / LEA protein-related	-0,80	2,18E-2
AT2G48140	protease inhibitor/seed storage/lipid transfer protein (LTP) family protein	-0,80	2,13E-2
AT5G23750	remorin family protein	-0,80	1,87E-2
AT1G54000	myrosinase-associated protein, putative	-0,80	1,87E-2
AT2G26370	MD-2-related lipid recognition domain-containing protein / ML domain-containing protein	-0,80	1,86E-2
AT3G19710	branched-chain amino acid aminotransferase, putative / branched-chain amino acid transaminase, putative (BCAT4)	-0,80	1,78E-2
AT4G24190	shepherd protein (SHD) / clavata formation protein, putative	-0,81	1,64E-2
AT2G37460	nodulin MtN21 family protein	-0,82	1,20E-2
AT4G26130	expressed protein	-0,82	1,18E-2
AT1G16400	cytochrome P450 family protein	-0,82	1,16E-2
AT1G03870	fasciclin-like arabinogalactan-protein (FLA9)	-0,82	1,04E-2
AT1G57590	pectinacetylesterase, putative	-0,82	1,01E-2
AT3G56000	glycosyl transferase family 2 protein	-0,83	9,05E-3
AT4G12420	multi-copper oxidase, putative (SKU5)	-0,83	8,96E-3
AT1G09760	U2 small nuclear ribonucleoprotein A, putative	-0,83	7,06E-3
AT4G37400	cytochrome P450 family protein	-0,83	6,82E-3
AT1G53830	pectinesterase family protein	-0,84	6,17E-3
AT4G17280	auxin-responsive family protein	-0,84	6,17E-3
AT5G42600	pentacyclic triterpene synthase, putative	-0,84	5,89E-3
AT5G22940	exostosin family protein	-0,84	5,41E-3
AT5G09980	expressed protein	-0,84	5,38E-3
AT2G14890	arabinogalactan-protein (AGP9)	-0,86	3,40E-3
AT1G09620	tRNA synthetase class I (I, L, M and V) family protein	-0,87	2,03E-3
AT5G23210	serine carboxypeptidase S10 family protein	-0,88	1,43E-3
AT3G01260	aldose 1-epimerase family protein	-0,88	1,37E-3
AT2G33830	dormancy/auxin associated family protein	-0,88	1,27E-3
AT4G12030	bile acid:sodium symporter family protein	-0,89	1,24E-3
AT3G49190	condensation domain-containing protein	-0,89	1,22E-3
AT3G44330	expressed protein	-0,89	1,19E-3
AT2G13550	expressed protein	-0,89	1,15E-3
AT4G19030	major intrinsic family protein / MIP family protein	-0,90	7,33E-4
AT1G17190	glutathione S-transferase, putative	-0,91	5,82E-4
AT4G16660	heat shock protein 70, putative / HSP70, putative	-0,91	5,42E-4
AT5G04120	phosphoglycerate/bisphosphoglycerate mutase family protein	-0,91	5,21E-4
AT3G58990	aconitase C-terminal domain-containing protein	-0,92	3,84E-4
AT4G31910	transferase family protein	-0,92	3,37E-4
AT2G34770	fatty acid hydroxylase (FAH1)	-0,94	1,93E-4
AT1G45015	MD-2-related lipid recognition domain-containing protein / ML domain-containing protein	-0,95	1,21E-4
AT5G41080	glycerophosphoryl diester phosphodiesterase family protein	-0,95	1,10E-4
AT1G78860	curculin-like (mannose-binding) lectin family protein	-0,95	1,00E-4
AT3G51280	male sterility MS5, putative	-0,96	8,92E-5
AT1G12080	expressed protein	-0,96	7,29E-5
AT5G08330	TCP family transcription factor, putative	-0,97	6,83E-5
AT4G28440	DNA-binding protein-related	-0,97	6,76E-5
AT3G48340	cysteine proteinase, putative	-0,99	2,71E-5
AT4G30290	xyloglucan:xyloglucosyl transferase, putative / xyloglucan endotransglycosylase, putative / endo-xyloglucan transferase, putative	-1,03	6,69E-6
AT5G53250	arabinogalactan-protein, putative (AGP22)	-1,03	5,41E-6
AT5G07990	flavonoid 3prim-monooxygenase/flavonoid 3prim-hydroxylase (F3primH)/cytochrome P450 75B1 (CYP75B1)/transparent testa 7 prote	-1,04	4,24E-6
AT5G10430	arabinogalactan-protein (AGP4)	-1,04	3,57E-6
AT5G25090	plastocyanin-like domain-containing protein	-1,08	8,48E-7
AT2G40080	ELF4 (EARLY FLOWERING 4)	-1,08	6,64E-7
AT4G27400	late embryogenesis abundant protein-related / LEA protein-related	-1,12	1,16E-7
AT3G06460	GNS1/SUR4 membrane family protein	-1,16	1,97E-8
AT2G20750	beta-expansin, putative (EXPB1)	-1,19	4,85E-9
AT4G33625	expressed protein	-1,23	8,25E-10
AT1G29020	calcium-binding EF hand family protein	-1,25	3,82E-10
AT4G02290	glycosyl hydrolase family 9 protein	-1,25	2,97E-10
AT3G05900	neurofilament protein-related	-1,28	8,98E-11
AT2G25150	transferase family protein	-1,28	6,73E-11
AT4G28250	beta-expansin, putative (EXPB3)	-1,29	4,49E-11
AT3G25190	nodulin, putative	-1,37	0,00E+0
AT2G33790	pollen Ole e 1 allergen and extensin family protein	-1,38	0,00E+0
AT5G42590	cytochrome P450 71A16, putative (CYP71A16)	-1,42	0,00E+0
AT2G18800	xyloglucan:xyloglucosyl transferase, putative / xyloglucan endotransglycosylase, putative / endo-xyloglucan transferase, putative	-1,44	0,00E+0
AT4G25250	invertase/pectin methylesterase inhibitor family protein	-1,56	0,00E+0
AT1G64390	endo-1,4-beta-glucanase, putative / cellulase, putative	-1,60	0,00E+0
AT4G35100	plasma membrane intrinsic protein (SIMIP)	-1,61	0,00E+0
AT4G01630	expansin, putative (EXP17)	-1,76	0,00E+0
AT3G18000	phosphoethanolamine N-methyltransferase 1 / PEAMT 1 (NMT1)	-1,78	0,00E+0
