# Supplementary figures and images for: Identification of genes involved in the ACC-mediated control of root cell elongation in Arabidopsis thaliana
Source: BMC Plant Biol. 2012 Nov 7;12:208. doi: 10.1186/1471-2229-12-208 (PMC3502322; doi:10.1186/1471-2229-12-208)

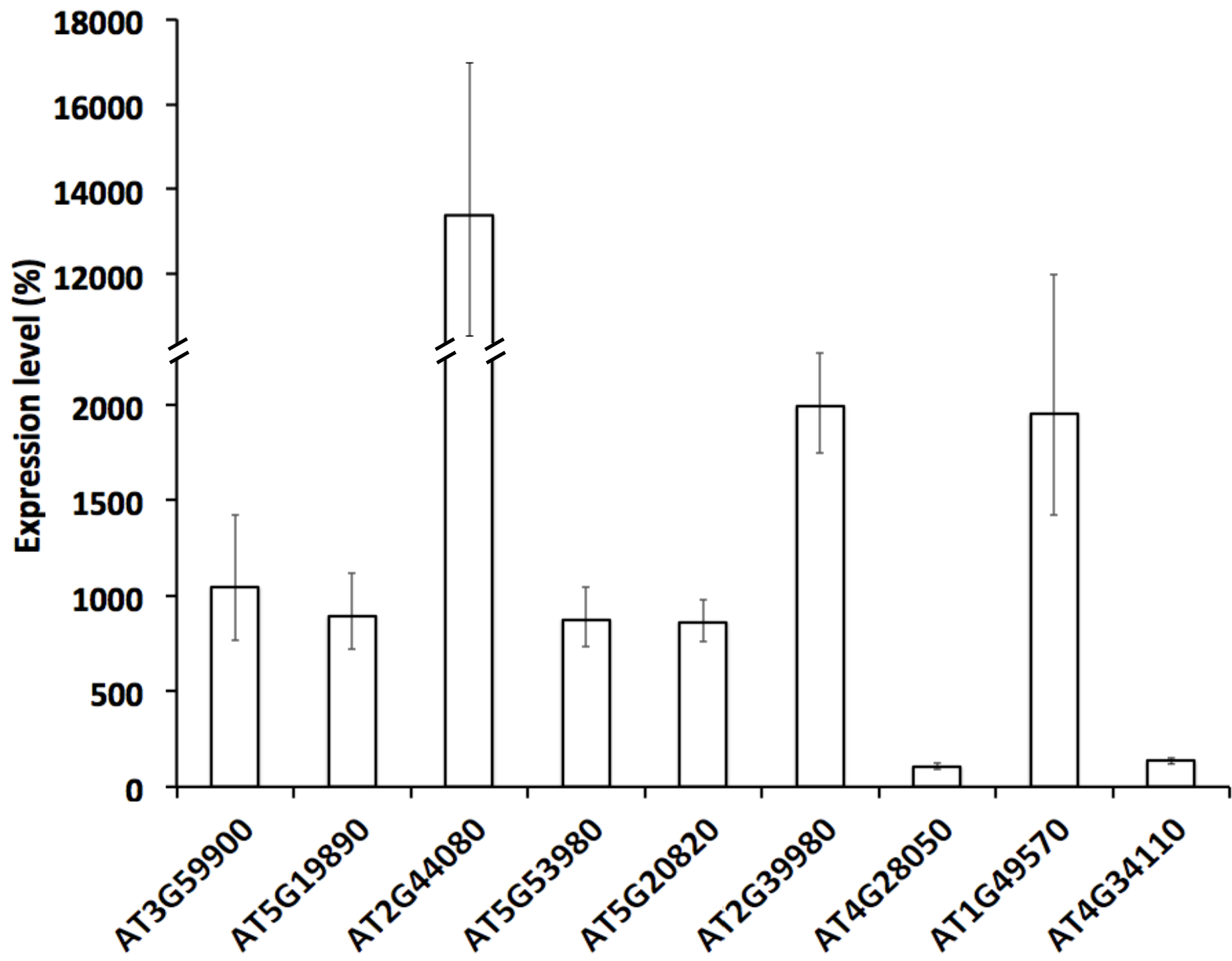

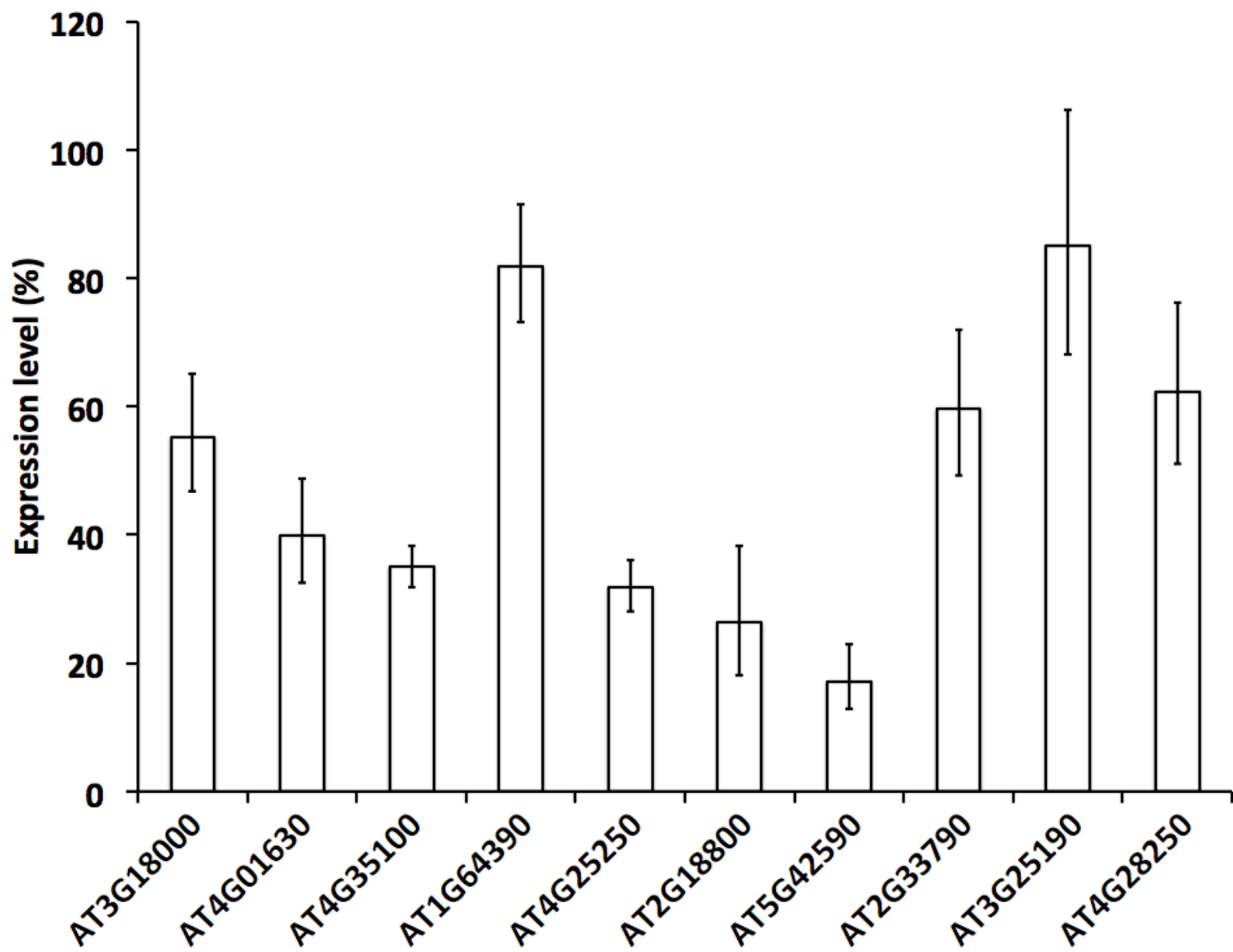

Supplement: Additional file 2 — qPCR analysis of the 10 most up and down regulated genes upon 3 hr 5μM ACC addition to Arabidopsis roots. Expression is presented as relative % towards the gene’s expression under control conditions. [file 1471-2229-12-208-S2.pdf]
