# Supplementary material for: Identification of genes involved in the ACC-mediated control of root cell elongation in Arabidopsis thaliana
Source: BMC Plant Biol. 2012 Nov 7;12:208. doi: 10.1186/1471-2229-12-208 (PMC3502322; doi:10.1186/1471-2229-12-208)
Supplement: Additional file 3 — Expression profiles in the root of the genes presented in Table 2. Data were extracted from the Arabidopsis eFP browser. A) ethylene-related genes, B) auxin-related genes, C) AGPs and HRGPs, D) peroxidases, E) 10 most up regulated genes, F) 10 most down regulated genes. [file 1471-2229-12-208-S3.pdf]

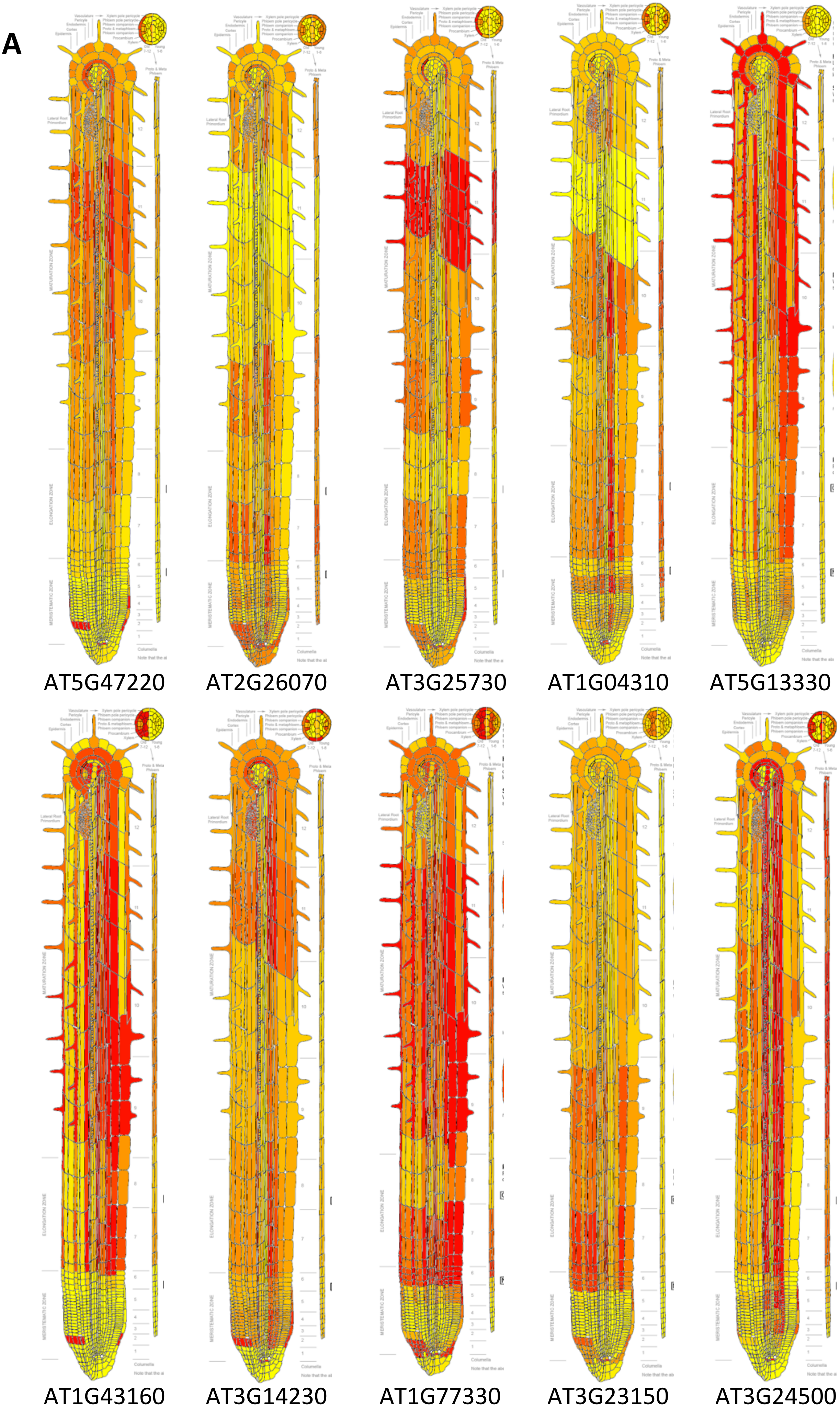
A
AT5G47220
AT2G26070
AT3G25730
AT1G04310
AT5G13330
AT1G43160
AT3G14230
AT1G77330
AT3G23150
AT3G24500

B

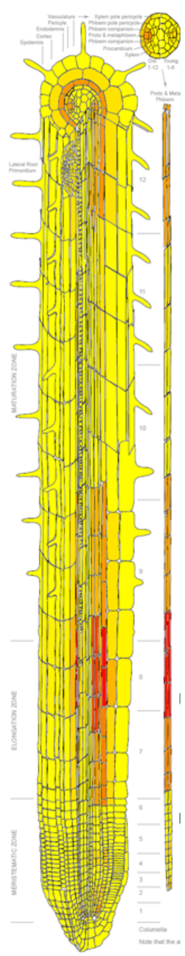
AT5G208200

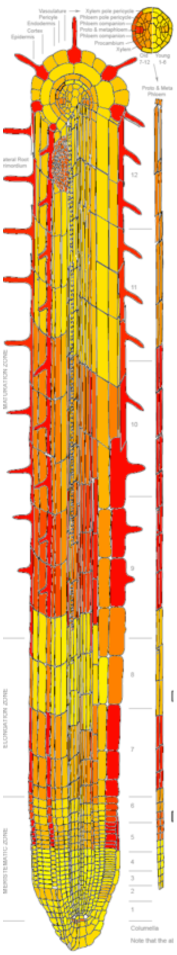
AT5G54490

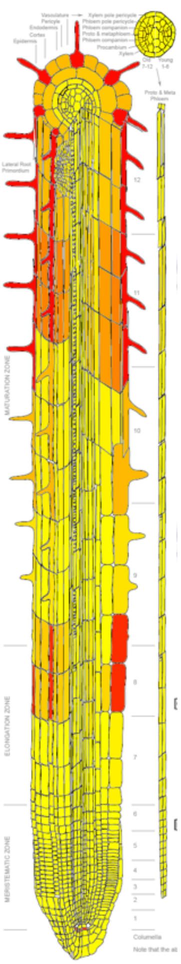
AT4G37390

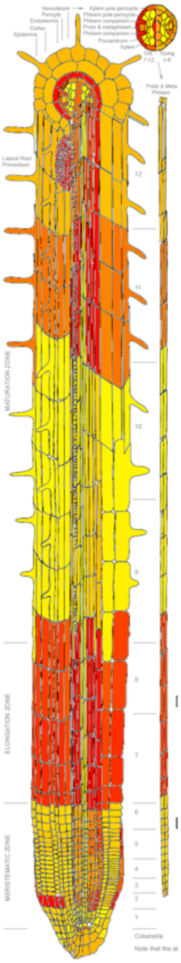
AT3G23030

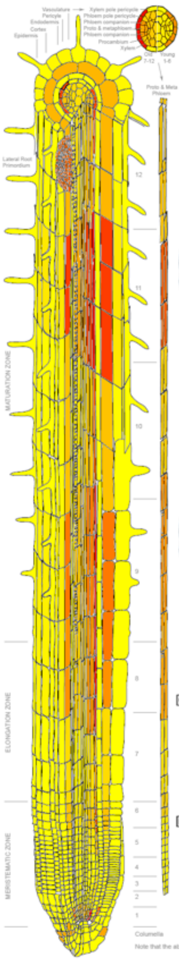
AT1G48690

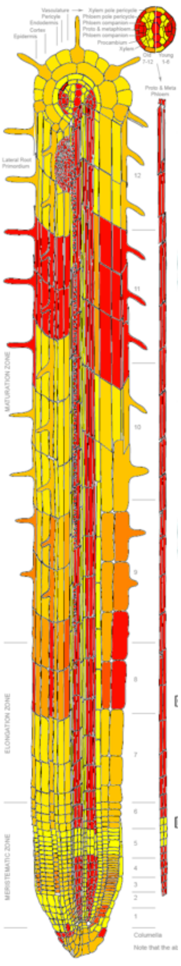
AT2G33830

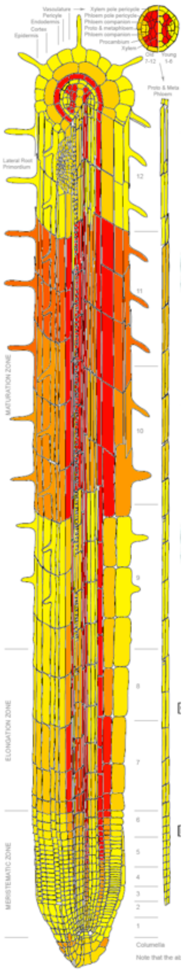
AT4G17280

C

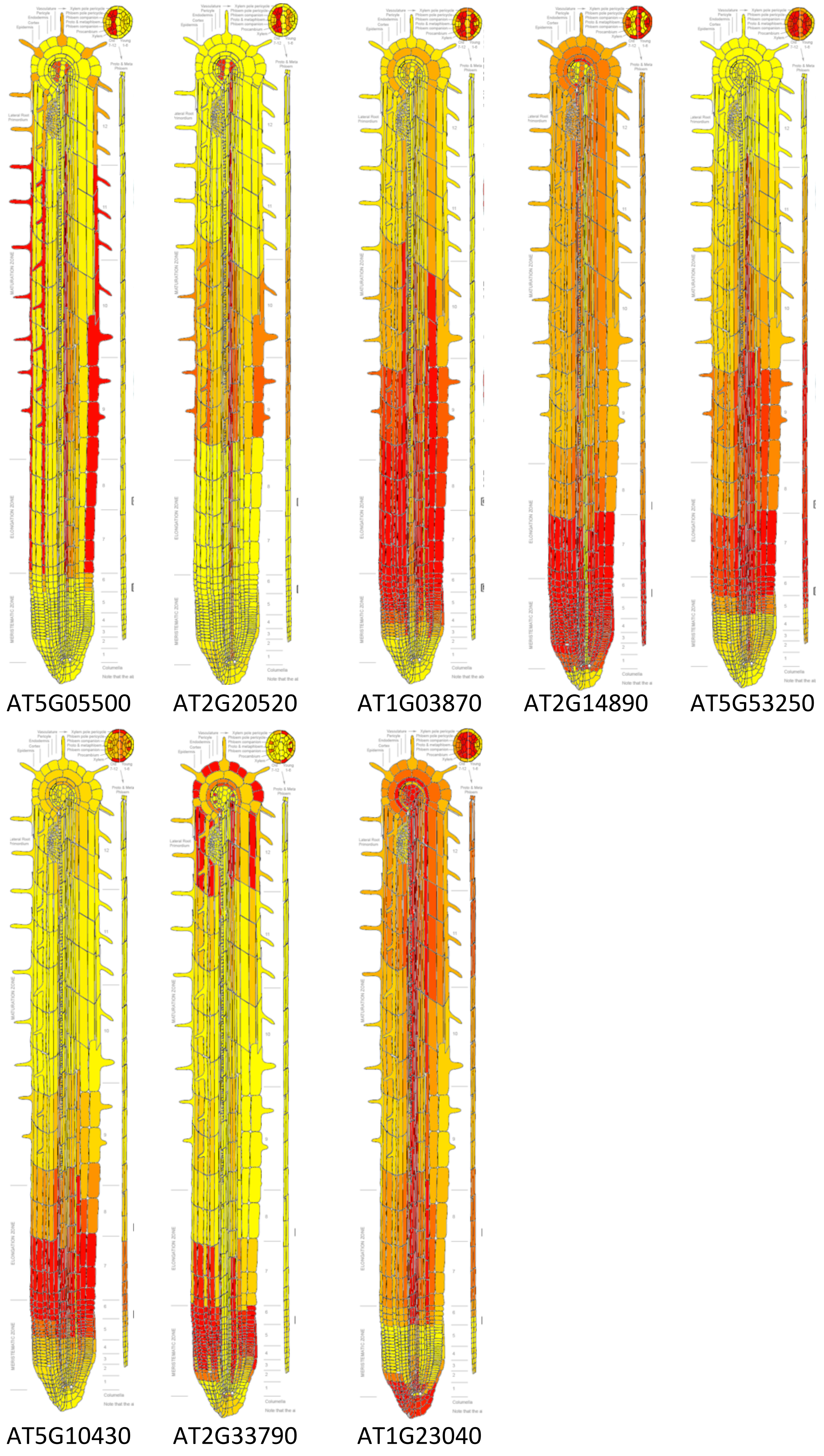
AT5G05500
AT2G20520
AT1G03870
AT2G14890
AT5G53250
AT5G10430
AT2G33790
AT1G23040

D

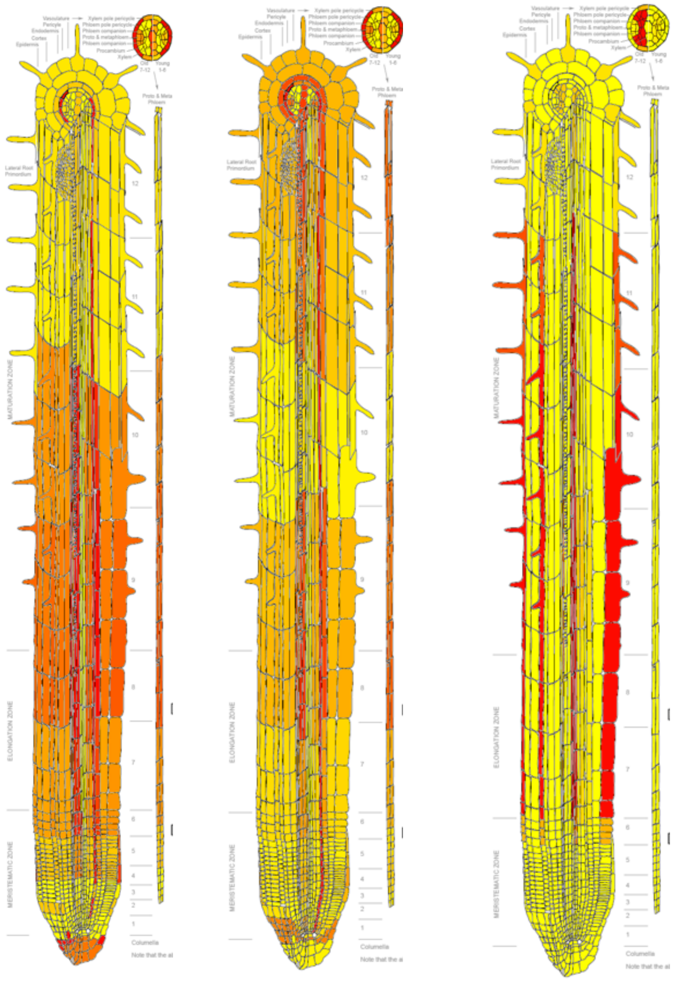

AT5G19890 AT1G49570 AT3G49960

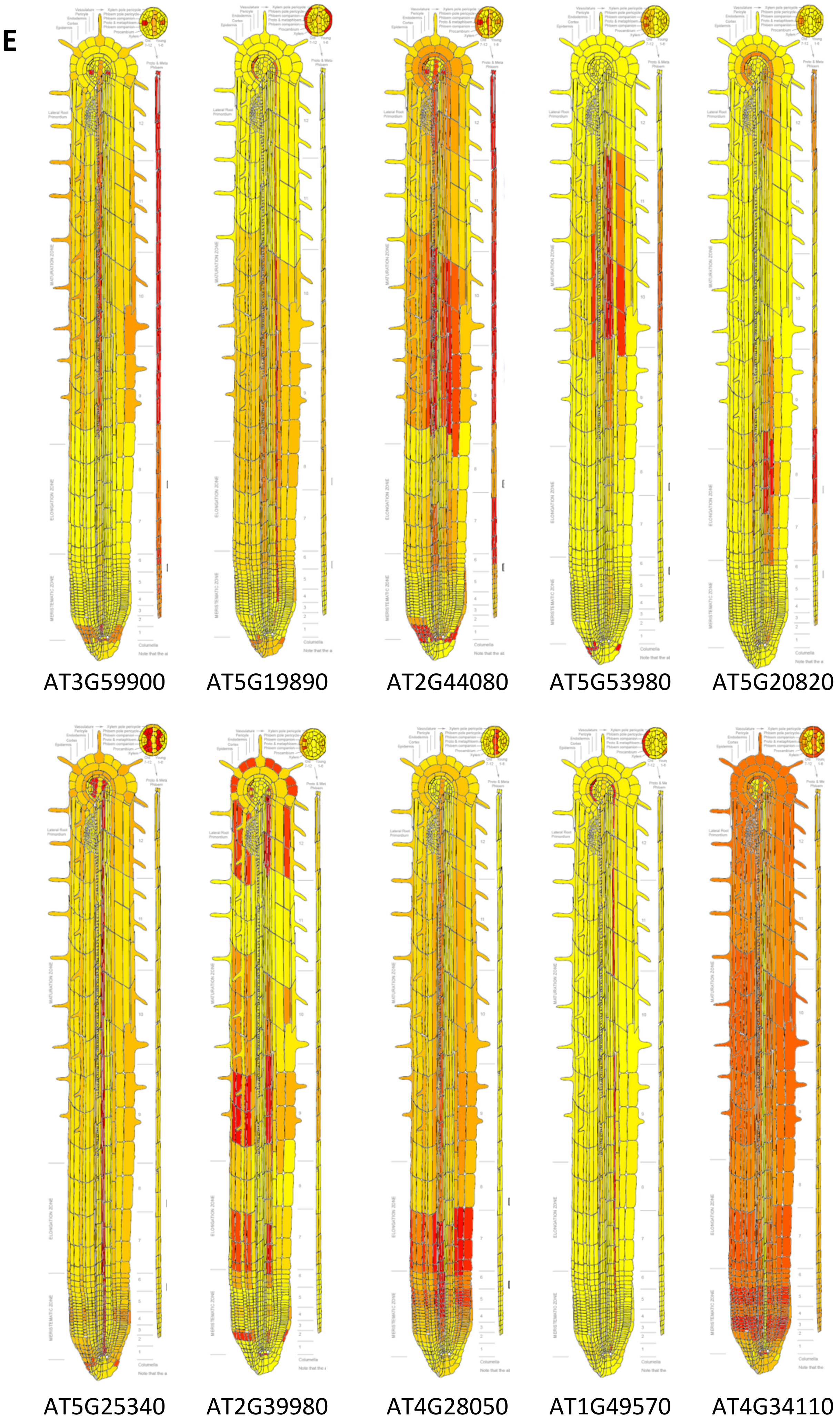
E
AT3G59900
AT5G19890
AT2G44080
AT5G53980
AT5G20820
AT5G25340
AT2G39980
AT4G28050
AT1G49570
AT4G34110

F

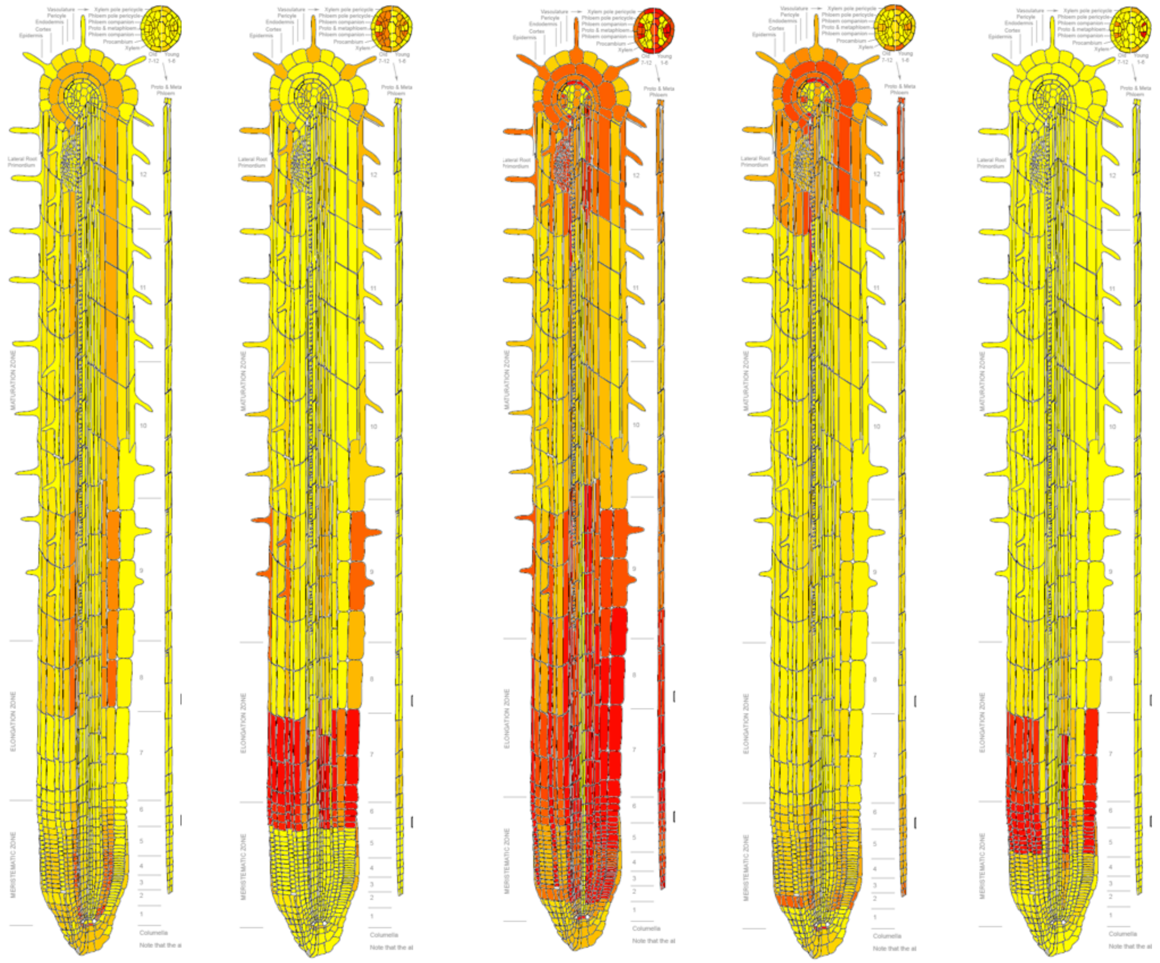

AT3G18000 AT4G01630 AT4G35100 AT1G64390 AT4G25250

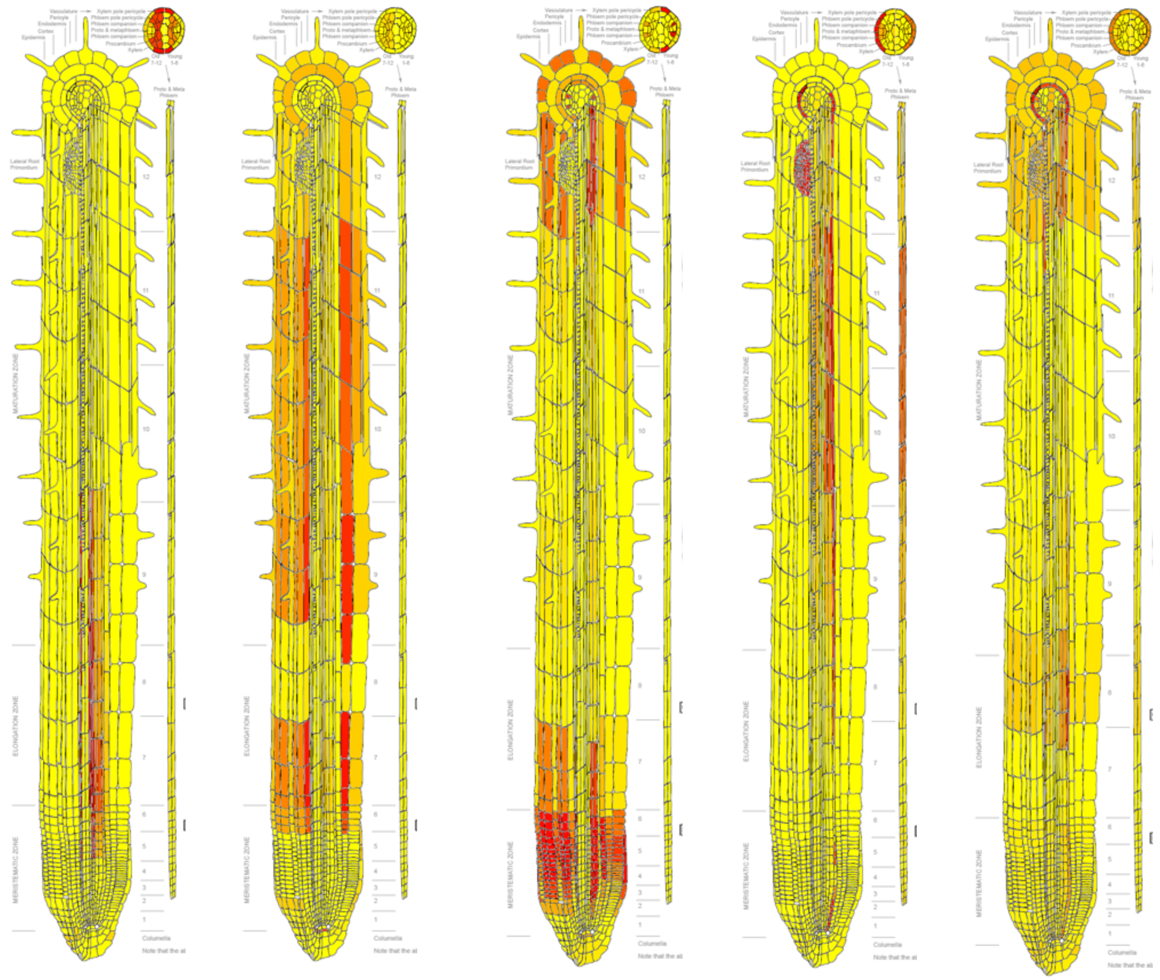

AT2G18800 AT5G42590 AT2G33790 AT3G25190 AT4G28250
