# Supplementary material for: Identification of genes involved in the ACC-mediated control of root cell elongation in Arabidopsis thaliana
Source: BMC Plant Biol. 2012 Nov 7;12:208. doi: 10.1186/1471-2229-12-208 (PMC3502322; doi:10.1186/1471-2229-12-208)
Supplement: Additional file 4 — Enriched Gene Ontology (GO) terms in the differentially expressed genes. A) cluster of up regulated genes, B) cluster of down regulated genes, with the legend linking the numbers to the GI terms. [file 1471-2229-12-208-S4.pdf]

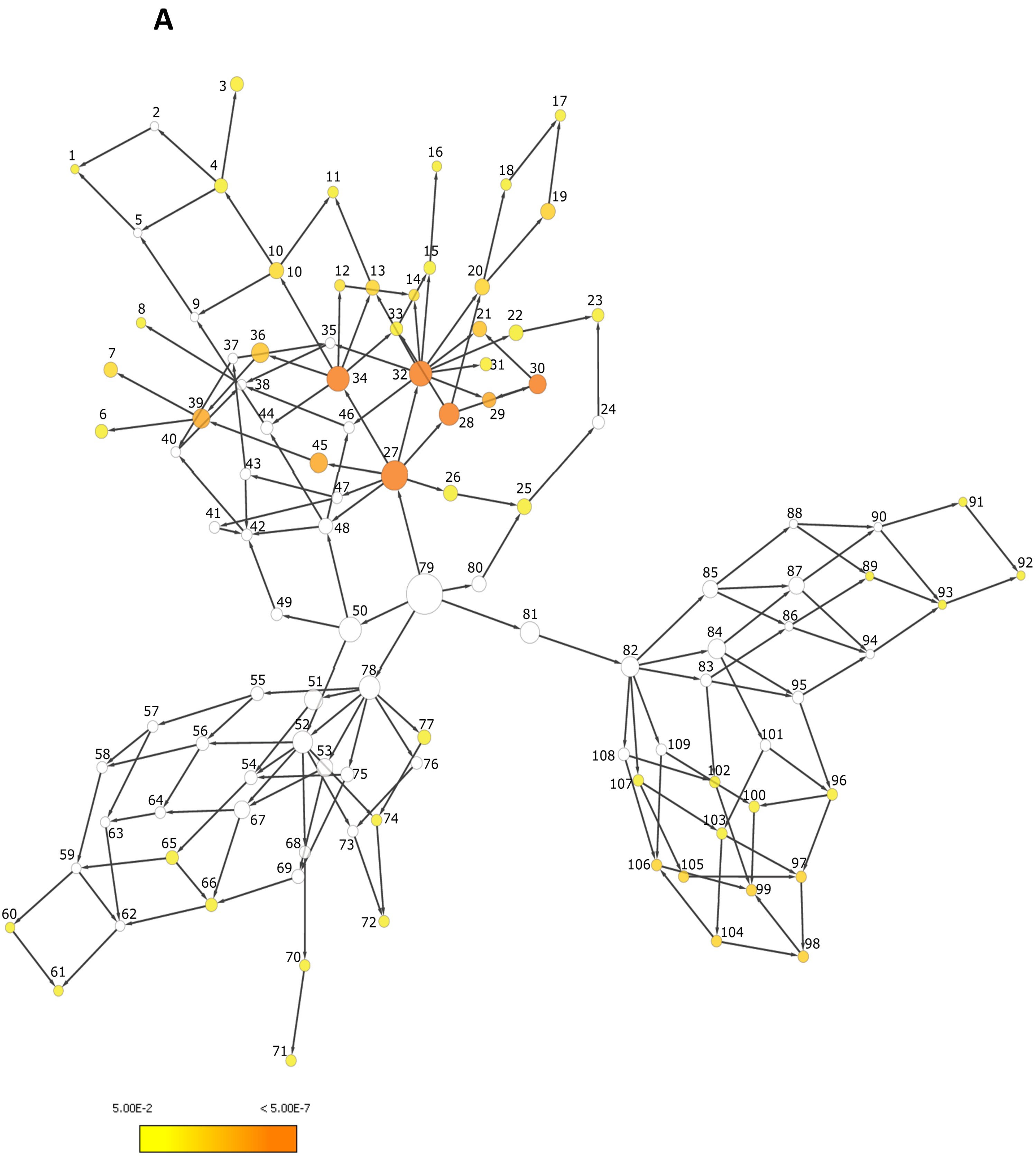
A
5.00E-2
< 5.00E-7

**B**

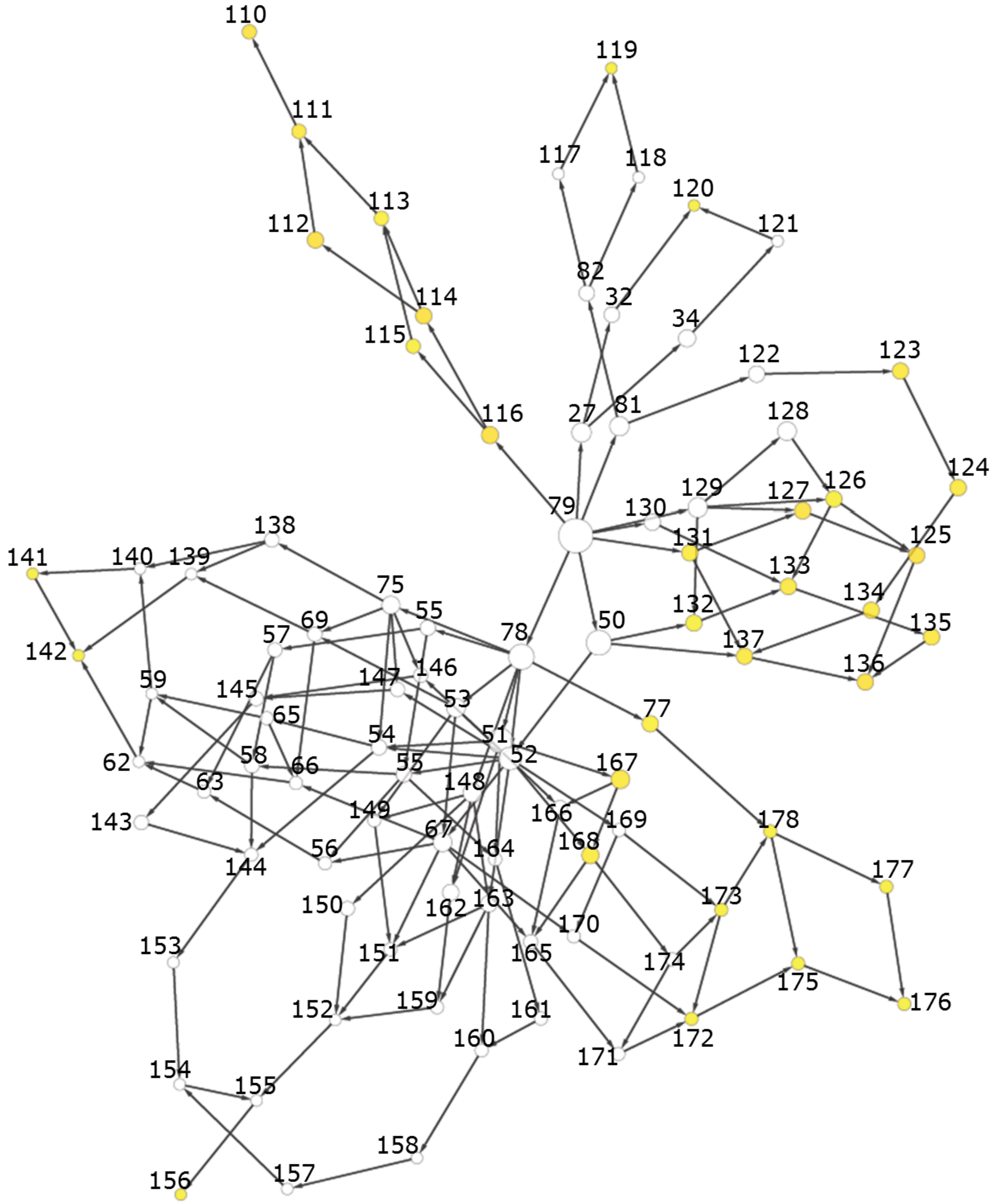
110
111
112
113
114
115
116
119
117
118
120
121
82
32
34
122
123
27
81
128
124
129
127
126
79
130
131
125
133
134
138
141
140
139
75
55
132
50
135
69
57
78
137
136
142
146
59
147
145
53
77
65
54
51
52
167
62
58
66
55
63
148
166
169
149
178
143
67
168
164
56
144
177
173
150
162
163
170
151
153
165
174
175
159
152
161
176
160
172
171
154
155
158
157
156

| | |
|---|---|
| 1. cellular response to selenium ion | 2. response to selenium ion |
| 3. response to cadmium ion | 4. response to metal ion |
| 5. cellular response to metal ion | 6. response to auxin stimulus |
| 7. response to abscisic acid stimulus | 8. cellular response to sulfate starvation |
| 9. cellular response to inorganic substance | 10. response to inorganic substance |
| 11. response to reactive oxygen species | 12. response to oxygen levels |
| 13. response to oxidative stress | 14. response to hypoxia |
| 15. response to water deprivation | 16. response to desiccation |
| 17. hyperosmotic salinity response | 18. hyperosmotic response |
| 19. response to salt stress | 20. response to osmotic stress |
| 21. response to cold | 22. defense response |
| 23. defense response to bacterium | 24. response to bacterium |
| 25. response to other organism | 26. response to biotic stimulus |
| 27. response to stimulus | 28. response to abiotic stimulus |
| 29. response to heat | 30. response to temperature stimulus |
| 31. response to wounding | 32. response to stress |
| 33. response to water | 34. response to chemical stimulus |
| 35. response to starvation | 36. response to organic substance |
| 37. response to nutrient levels | 38. cellular response to starvation |
| 39. response to hormone stimulus | 40. cellular response to nutrient levels |
| 41. cellular response to external stimulus | 42. cellular response to extracellular stimulus |
| 43. response to extracellular stimulus | 44. cellular response to chemical stimulus |
| 45. response to endogenous stimulus | 46. cellular response to stress |
| 47. response to external stimulus | 48. cellular response to stimulus |
| 49. cell communication | 50. cellular process |
| 51. primary metabolic process | 52. cellular metabolic process |
| 53. biosynthetic process | 54. cellular amino acid and derivative metabolic process |
| 55. nitrogen compound metabolic process | 56. cellular nitrogen compound metabolic process |
| 57. amine metabolic process | 58. cellular amine metabolic process |
| 59. cellular biogenic amine metabolic process | 60. polyamine metabolic process |
| 61. polyamine biosynthetic process | 62. cellular biogenic amine biosynthetic process |
| 63. amine biosynthetic process | 64. cellular nitrogen compound biosynthetic process |
| 65. cellular amino acid derivative metabolic process | 66. cellular amino acid derivative biosynthetic process |
| 67. cellular biosynthetic process | 68. generation of precursor metabolites and energy |
| 69. small molecule biosynthetic process | 70. energy derivation by oxidation of organic compounds |
| 71. cellular respiration | 72. toxin catabolic process |
| 73. cellular catabolic process | 74. toxin metabolic process |
| 75. small molecule metabolic process | 76. catabolic process |
| 77. secondary metabolic process | 78. metabolic process |
| 79. biological process | 80. multi-organism process |
| 81. biological regulation | 82. regulation of biological process |
| 83. negative regulation of biological process | 84. regulation of cellular process |
| 85. regulation of metabolic process | 86. negative regulation of metabolic process |
| 87. regulation of cellular metabolic process | 88. regulation of catabolic process |
| 89. negative regulation of catabolic process | 90. regulation of cellular catabolic process |
| 91. regulation of autophagy | 92. negative regulation of autophagy |

| | |
|---|---|
| 93. negative regulation of cellular catabolic process | 94. negative regulation of cellular metabolic process |
| 95. negative regulation of cellular process | 96. negative regulation of cell communication |
| 97. negative regulation of signal transduction | 98. negative regulation of two-component signal transduction system (phosphorelay) |
| 99. negative regulation of ethylene mediated signaling pathway | 100. negative regulation of signaling pathway |
| 101. regulation of cell communication | 102. negative regulation of response to stimulus |
| 103. regulation of signal transduction | 104. regulation of two-component signal transduction system (phosphorelay) |
| 105. negative regulation of signaling process | 106. regulation of ethylene mediated signaling pathway |
| 107. regulation of signaling process | 108. regulation of response to stimulus |
| 109. regulation of signaling pathway | 110. plant-type cell wall loosening |
| 111. plant-type cell wall modification | 112. cell wall modification |
| 113. plant-type cell wall organization | 114. cell wall organization |
| 115. plant-type cell wall organization or biogenesis | 116. cell wall organization or biogenesis |
| 117. regulation of circadian rhythm | 118. positive regulation of biological |
| 119. positive regulation of circadian rhythm | 120. response to herbicide |
| 121. response to toxin | 122. regulation of biological quality |
| 123. regulation of anatomical structure size | 124. regulation of cellular component size |
| 125. developmental growth involved in morphogenesis | 126. anatomical structure morphogenesis |
| 127. developmental growth | 128. anatomical structure development |
| 129. developmental process | 130. cellular component organization |
| 131. growth | 132. cellular developmental process |
| 133. cellular component morphogenesis | 134. regulation of cell size |
| 135. cell morphogenesis | 136. unidimensional cell growth |
| 137. cell growth | 138. alcohol metabolic process |
| 139. alcohol biosynthetic process | 140. ethanolamine and derivative metabolic process |
| 141. choline metabolic process | 142. choline biosynthetic process |
| 143. carboxylic acid metabolic process | 144. cellular amino acid metabolic process |
| 145. oxoacid metabolic process | 146. organic acid metabolic process |
| 147. cellular ketone metabolic process | 148. macromolecule metabolic process |
| 149. macromolecule biosynthetic process | 150. gene expression |
| 151. cellular macromolecule biosynthetic process | 152. translation |
| 153. amino acid activation | 154. tRNA aminoacylation |
| 155. tRNA aminoacylation for protein translation | 156. leucyl-tRNA aminoacylation |
| 157. tRNA metabolic process | 158. ncRNA metabolic process |
| 159. cellular protein metabolic process | 160. RNA metabolic process |
| 161. nucleic acid metabolic process | 162. protein metabolic process |
| 163. cellular macromolecule metabolic process | 164. nucleobase, nucleoside, nucleotide and nucleic acid metabolic process |
| 165. cellular carbohydrate biosynthetic process | 166. carbohydrate biosynthetic process |
| 167. carbohydrate metabolic process | 168. cellular carbohydrate metabolic process |
| 169. sulfur metabolic process | 170. sulfur compound biosynthetic process |
| 171. glycoside biosynthetic process | 172. S-glycoside biosynthetic process |

| | |
|---|---|
| 173. S-glycoside metabolic process | 174. glycoside metabolic process |
| 175. glycosinolate biosynthetic process | 176. glucosinolate biosynthetic process |
| 177. glucosinolate metabolic process | 178. glycosinolate metabolic process |
